# Polygenic risk score with *KLK3* SNP-SNP interaction pairs for predicting prostate cancer aggressiveness

**DOI:** 10.1038/s43856-026-01645-z

**Published:** 2026-05-28

**Authors:** Hui-Yi Lin, Indrani Sarkar, Harun Mazumder, Po-Yu Huang, Kenneth R. Muir, Johanna Schleutker, Nora Pashayan, Jyotsna Batra, David E. Neal, Henrik Grönberg, Sune F. Nielsen, Børge G. Nordestgaard, Catherine M. Tangen, Robert J. MacInnis, Alicja Wolk, Demetrius Albanes, Ruth C. Travis, Janet L. Stanford, Lorelei A. Mucci, Adam S. Kibel, Olivier Cussenot, Sonja I. Berndt, Stella Koutros, Karina Dalsgaard Sørensen, Cezary Cybulski, Eli Marie Grindedal, Josef Hoegel, Christiane Maier, Robert J. Hamilton, Barry S. Rosenstein, Ana Vega, Manolis Kogevinas, Fredrik Wiklund, Kathryn L. Penney, Manuel R. Teixeira, Hermann Brenner, Esther M. John, Radka Kaneva, Christopher J. Logothetis, Susan L. Neuhausen, Kim De Ruyck, Piet Ost, Marija Gamulin, Nawaid Usmani, Frank Claessens, Jose Esteban Castelao, Paul A. Townsend, UKGPCS collaborators, Kenneth R. Muir, Zsofia Kote-Jarai, Rosalind A. Eeles, APCB BioResource (Australian Prostate Cancer BioResource), Jyotsna Batra, Zsofia Kote-Jarai, Christopher A. Haiman, Rosalind A. Eeles, the PRACTICAL consortium, Hui-Yi Lin, Kenneth R. Muir, Johanna Schleutker, Nora Pashayan, Jyotsna Batra, David E. Neal, Henrik Grönberg, Sune F. Nielsen, Børge G. Nordestgaard, Catherine M. Tangen, Robert J. MacInnis, Alicja Wolk, Demetrius Albanes, Ruth C. Travis, Janet L. Stanford, Lorelei A. Mucci, Adam S. Kibel, Olivier Cussenot, Sonja I. Berndt, Stella Koutros, Karina Dalsgaard Sørensen, Cezary Cybulski, Eli Marie Grindedal, Josef Hoegel, Christiane Maier, Robert J. Hamilton, Barry S. Rosenstein, Ana Vega, Manolis Kogevinas, Fredrik Wiklund, Kathryn L. Penney, Manuel R. Teixeira, Hermann Brenner, Esther M. John, Radka Kaneva, Christopher J. Logothetis, Susan L. Neuhausen, Piet Ost, Marija Gamulin, Nawaid Usmani, Frank Claessens, Jose Esteban Castelao, Paul A. Townsend, Zsofia Kote-Jarai, Christopher A. Haiman, Rosalind A. Eeles, Kim De Ruyck, Jong Y. Park, Jong Y. Park

**Affiliations:** 1 Biostatistics and Data Science Program, School of Public Health, Louisiana State University Health Sciences Center, New Orleans, LA USA; 2 Information and Communications Research Laboratories, Industrial Technology Research Institute, Hsinchu, Taiwan; 3 Division of Population Health, Health Services Research and Primary Care, University of Manchester, Oxford Road, Manchester, UK; 4 Institute of Biomedicine, University of Turku, Turku, Finland; 5 Genomics, Laboratory Division, Turku University Hospital, Turku, Finland; 6 Department of Public Health & Primary Care, Strangeways Research Laboratory, Worts Causeway, Cambridge, UK; 7 Department of Applied Health Research, University College London, London, UK; 8 School of Biomedical Sciences, Faculty of Health, Queensland University of Technology, Brisbane, QLD Australia; 9 Centre for Genomics and Personalised Health, Queensland University of Technology, Brisbane, QLD Australia; 10 Translational Research Institute, QUT, Woolloongabba, Brisbane, QLD Australia; 11 Nuffield Department of Surgical Sciences, University of Oxford, Oxford, UK; 12 Department of Oncology, University of Cambridge, Cambridge, UK; 13 Cancer Research UK, Li Ka Shing Centre, Cambridge Research Institute, Cambridge, UK; 14 Department of Medical Epidemiology and Biostatistics, Karolinska Institutet, Stockholm, Sweden; 15 Department of Clinical Biochemistry, Herlev and Gentofte Hospital, Copenhagen University Hospital, Herlev, Denmark; 16 Faculty of Health and Medical Sciences, University of Copenhagen, Copenhagen, Denmark; 17 SWOG Statistical Center, Fred Hutchinson Cancer Research Center, Seattle, WA USA; 18 Cancer Epidemiology Division, Cancer Council Victoria, East Melbourne, VIC Australia; 19 Centre for Epidemiology and Biostatistics, Melbourne School of Population and Global Health, The University of Melbourne, Parkville, VIC Australia; 20 Institute of Environmental Medicine, Karolinska Institutet, Stockholm, Sweden; 21 Division of Cancer Epidemiology and Genetics, National Cancer Institute, NIH, Bethesda, MD USA; 22 Cancer Epidemiology Unit, Nuffield Department of Population Health, University of Oxford, Oxford, UK; 23 Division of Public Health Sciences, Fred Hutchinson Cancer Research Center, Seattle, WA USA; 24 Department of Epidemiology, School of Public Health, University of Washington, Seattle, WA USA; 25 Department of Epidemiology, Harvard T. H. Chan School of Public Health, Boston, MA USA; 26 Division of Urologic Surgery, Brigham and Women’s Hospital, Boston, MA USA; 27 Sorbonne Universite, Tenon Hospital, Paris, France; 28 CeRePP, Tenon Hospital, Paris, France; 29 Department of Molecular Medicine, Aarhus University Hospital, Aarhus N, Denmark; 30 Department of Clinical Medicine, Aarhus University, Aarhus N, Denmark; 31 International Hereditary Cancer Center, Department of Genetics and Pathology, Pomeranian Medical University, Szczecin, Poland; 32 Department of Medical Genetics, Oslo University Hospital, Oslo, Norway; 33 Institute for Human Genetics, University Hospital Ulm, Ulm, Germany; 34 Humangenetik Tuebingen, Tuebingen, Germany; 35 Dept. of Surgical Oncology, Princess Margaret Cancer Centre, Toronto, ON Canada; 36 Dept. of Surgery (Urology), University of Toronto, Toronto, ON Canada; 37 Department of Radiation Oncology and Department of Genetics and Genomic Sciences, Icahn School of Medicine at Mount Sinai, New York, NY USA; 38 Fundación Pública Galega Medicina Xenómica, Santiago de Compostela, Spain; 39 Instituto de Investigación Sanitaria de Santiago de Compostela, Santiago de Compostela, Spain; 40 Centro de Investigación en Red de Enfermedades Raras (CIBERER), Madrid, Spain; 41 ISGlobal, Barcelona, Spain; 42 IMIM (Hospital del Mar Medical Research Institute), Barcelona, Spain; 43 Universitat Pompeu Fabra (UPF), Barcelona, Spain; 44 CIBER Epidemiología Salud Pública (CIBERESP), Madrid, Spain; 45 Channing Division of Network Medicine, Department of Medicine, Brigham and Women’s Hospital/Harvard Medical School, Boston, MA USA; 46 Department of Laboratory Genetics, Portuguese Oncology Institute of Porto (IPO Porto)/Porto Comprehensive Cancer Center, Porto, Portugal; 47 Cancer Genetics Group, IPO Porto Research Center (CI-IPOP)/RISE@CI-IPOP (Health Research Network), Portuguese Oncology Institute of Porto (IPO Porto) / Porto Comprehensive Cancer Center, Porto, Portugal; 48 School of Medicine and Biomedical Sciences (ICBAS), University of Porto, Porto, Portugal; 49 Division of Clinical Epidemiology and Aging Research, German Cancer Research Center (DKFZ), Heidelberg, Germany; 50 German Cancer Consortium (DKTK), German Cancer Research Center (DKFZ), Heidelberg, Germany; 51 Departments of Epidemiology & Population Health and of Medicine, Division of Oncology, Stanford Cancer Institute, Stanford University School of Medicine, Stanford, CA USA; 52 Molecular Medicine Center, Department of Medical Chemistry and Biochemistry, Medical University of Sofia, Sofia, Bulgaria; 53 The University of Texas M. D. Anderson Cancer Center, Department of Genitourinary Medical Oncology, Houston, TX USA; 54 Department of Population Sciences, Beckman Research Institute of the City of Hope, Duarte, CA USA; 55 Ghent University, Faculty of Medicine and Health Sciences, Basic Medical Sciences, Gent, Belgium; 56 Department of Radiotherapy, Ghent University Hospital, Gent, Belgium; 57 Department of Oncology, University Hospital Centre Zagreb, University of Zagreb, School of Medicine, Zagreb, Croatia; 58 Department of Oncology, Cross Cancer Institute, University of Alberta, Edmonton, AB Canada; 59 Division of Radiation Oncology, Cross Cancer Institute, Edmonton, AB Canada; 60 Department of Cellular and Molecular Medicine, Molecular Endocrinology Laboratory, KU Leuven, Belgium; 61 Genetic Oncology Unit, CHUVI Hospital, Complexo Hospitalario Universitario de Vigo, Instituto de Investigación Biomédica Galicia Sur (IISGS), Vigo (Pontevedra), Spain; 62 Division of Cancer Sciences, Manchester Cancer Research Centre, Faculty of Biology, Medicine and Health, Manchester Academic Health Science Centre, NIHR Manchester Biomedical Research Centre, Health Innovation Manchester, University of Manchester, Manchester, UK; 63 The University of Surrey, Guildford, Surrey, UK; 64 The Institute of Cancer Research, London, UK; 65 Center for Genetic Epidemiology, Department of Preventive Medicine, Keck School of Medicine, University of Southern California/Norris Comprehensive Cancer Center, Los Angeles, CA USA; 66 Royal Marsden NHS Foundation Trust, London, UK; 67 Department of Cancer Epidemiology, Moffitt Cancer Center, Tampa, FL USA

**Keywords:** Cancer genetics, Cancer models

## Abstract

**Background:**

Prostate cancer (PCa) is heterogeneous, making risk stratification essential for clinical care. Although polygenic risk scores (PRSs) with main effects of single-nucleotide polymorphisms (SNPs) can help identify individuals at high risk before biological and clinical onset, a PRS for predicting PCa aggressiveness remains underdeveloped. The *KLK3*, which encodes prostate-specific antigen (PSA), is linked to PCa aggressiveness. Recent findings on *KLK3* SNP-SNP interactions show promise for predicting PCa aggressiveness. The objective of this study is to develop a PRS (PRS-KLK3int) by examining *KLK3* SNP-SNP interaction pairs.

**Methods:**

The PRS-KLK3int was developed based on a discovery set (10,836 PCa patients) and two validation sets with 14,348 and 16,584 patients of European ancestry. A total of 3145 SNP pairs and two published PRSs were evaluated.

**Results:**

This study developed a PRS-KLK3int with 284 SNPs, combining an existing PRS with 270 SNPs and 12 SNP-SNP interaction pairs with 15 SNPs (one overlapped). All these 12 pairs were involved with at least one SNP from *KLK3*. The PRS-KLK3int outperformed two existing PRSs in predicting PCa aggressiveness (p-values: 3.5×10^−18^, 9×10^−14^, and 1.7×10^−20^ for the three sets). It effectively distinguished high-risk from low-risk groups across all datasets. The top 1% high-risk group had a higher prevalence of PCa aggressiveness than the middle 50% group (45.5% vs. 25.9%, OR = 2.38, p = 2.2×10^−5^) in the discovery set, and similar results were observed in validation sets (OR = 2.56, p = 4.3×10^−6^; OR = 2.07, p = 2.1×10^−5^).

**Conclusions:**

These findings support PRS-KLK3int as a valuable tool for PCa severity stratification, especially in identifying extremely high-risk PCa patients.

## Plain language summary

Prostate cancer (PCa) can range from mild to very aggressive, so predicting severity early is important. Genetic tools called polygenic risk scores (PRS) can estimate risk before biological signs appear, helping doctors act sooner. Current PRSs don’t predict aggressiveness well. This study developed a new score, PRS-KLK3int, using genetic interactions in the KLK3 gene, which is linked to prostate-specific antigen (PSA) and cancer severity. Researchers analyzed data from over 10,000 patients and confirmed results in two large groups. PRS-KLK3int, built from 284 genetic markers, outperformed older scores in identifying aggressive cancer. PCa patients in the top 1% risk group were about twice as likely to have aggressive cancer. This tool could guide early screening and prevention before PCa progression starts.

## Abstract (web summary)

Lin et al. developed and validated a polygenic risk score (PRS-KLK3int) that incorporates SNP-SNP interactions involving KLK3 to predict prostate cancer aggressiveness. PRS-KLK3int consistently outperforms existing risk scores and can improve the identification of high-risk groups before the biological onset of aggressive disease.

## Introduction

Precise risk classification is essential for guiding treatment or monitoring decisions for prostate cancer (PCa) patients. As a heterogeneous disease, PCa accounts for 11% of all cancer deaths, making it the second leading cause of death in American men in 2024^1^. The risk of PCa and PCa-specific mortality rates are significantly different in different racial groups, likely due to a combination of environmental, biological, and genetic factors^2^. While the 5-year survival rate for localized and regional PCa is nearly 100%, that of PCa patients with distant metastasis is only 34%^3^. Due to the substantial clinical heterogeneity, distinguishing between indolent and aggressive PCa at diagnosis remains challenging^4^. Depending on the nature of selected biomarkers, risk stratification tools can be utilized at different stages of PCa disease development. Most current risk stratification tools target early detection of PCa aggressiveness in the biological or clinical onset stages. D’Amico Risk Classification and the Cancer of the Prostate Risk Assessment (CAPRA) Score are based on tumor characteristics (such as prostate-specific antigen (PSA), Gleason score, and clinical stage) for early detection in the clinical onset stage of PCa aggressiveness^5^. Gene expression signatures of tumors for PCa progression (such as Oncotype-DX^6,7^, and Decipher^7^) are suitable for early detection in both biological and clinical onset stages. However, few reliable risk stratification tools were reported to predict PCa aggressiveness before its biological onset^8^.

Genetics plays an important role in PCa development. A large-scale twin study has shown that genetics contributes to 57% of PCa development^9^. A handful of inherited genetic variants or single nucleotide polymorphisms (SNPs) for predicting PCa aggressiveness have been identified in genome-wide association studies (GWAS)^10,11^. Due to the limited predictive power of individual SNPs, many studies developed polygenic risk scores (PRSs), which are primarily calculated as a weighted sum of allele dosages from multiple GWAS-identified SNPs^12,13^. However, these conventional PRSs do not consider SNP-SNP interactions. Despite this limitation, PRSs are increasingly recognized as valuable tools in precision medicine for various phenotypes, including PCa.

While many PRSs have been developed to assess PCa risk, few specifically address PCa aggressiveness. One PRS with 290 SNPs predicts age at diagnosis of aggressive PCa, defined as with Gleason score ≥7, PSA  ≥  10 ng/mL, T3-T4 stage, nodal metastases, or distant metastases, based on ~72,000 men^14^. Another study developed a PRS of predicting age at PCa diagnosis with 166 SNPs using data from ~76,000 men^15^. Because age at PCa diagnosis is positively associated with PCa-specific mortality^16–18^, these two PRSs are related to PCa aggressiveness. However, these scores’ utility in predicting PCa aggressiveness may be limited as they were designed with a focus on age at PCa diagnosis rather than disease severity itself, and included both non-PCa men and PCa patients in developing these scores. Additionally, their focus on SNP main effects does not account for potential SNP-SNP interactions. This presents an opportunity for improvement.

SNP-SNP interaction is considered the key to improving prediction accuracy for PRSs. Our previous study identified 3145 SNP-SNP interaction pairs for predicting PCa aggressiveness. The majority (98.6%) of them involved *KLK3*^8^. *KLK3* encodes the PSA, which is used for PCa screening, diagnosis, and prognostic monitoring^19^. Increased levels of PSA in the bloodstream may suggest the existence of PCa or other prostate-related issues^20^. Previous studies showed that SNPs in *KLK3* were significantly associated with PSA levels, PCa risk, and aggressiveness^8,21–25^. Given the substantial role of *KLK3* in PCa biology and the prevalence of SNP-SNP interaction pairs relating to *KLK3*, we aim to develop and validate a polygenic risk score (PRS-KLK3int) that integrates these interactions. The objective of this study is to assess whether this PRS-KLK3int can improve the prediction of PCa aggressiveness compared to existing PRSs that are based exclusively on SNP main effects.

## Methods

### Statistics and reproducibility

The main statistical methods utilized in this study include the SNP Interaction Pattern Identifier (SIPI) approach, the RR3x3 method (Risk Ratio scoring based on 3 × 3 genotype pair combinations), and logistic models. The details and usage of these methods are listed in the following sections. For the study population, this study included 41,768 PCa patients with European ancestry and valid PCa aggressiveness data at the OncoArray cohort from the PRACTICAL consortium^8^. For this study, the SNP data in the PCa OncoArray cohort were used. For independent datasets, we excluded the PCa patients in our previous study^8^, which was used to identify the 3145 SNP interaction pairs. For enhancing the reproducibility of the proposed genetic risk scores, the participants are divided into 3 independent sets: the United Kingdom (UK), USA/Canada/Australia (UCA), and other European countries (EU) sets. We used the UK set as the discovery set (*n* = 10,836), the UCA set (*n* = 14,348), and the EU set (*n* = 16,584) as the two validation sets. PCa aggressiveness is defined as a Gleason score≥ 8, PSA ≥ 100 ng/mL, disease-distant metastasis stage at diagnosis, or death from PCa, consistent with our previous study^26^. This study was approved by the Louisiana State University Health Science Center Institutional Review Board under protocol number 5543. Patient consent was not required because deidentified data were used in this study.

### SNP data and quality controls

This study used both genotyped and imputed SNP data. The genotyped SNP data were generated using the Infinium OncoArray (Illumina, San Diego, CA). The imputed SNP data were generated based on the genotyped data using three panels of the 1000 Genomes Project as reference. Imputation was performed using SHAPEIT2 and IMPUTE2. One-step imputation has also been performed for regions surrounding known susceptibility loci. Strict quality control was applied for the OncoArray studies. SNPs were excluded if they had a call rate <95% and departed from Hardy-Weinberg equilibrium at *p* < 1 × 10^−12^ in PCa patients. Samples were excluded with a call rate of <95%. Ancestry analysis used a principal component analysis based on 2318 ancestry informative markers. The details of population stratification and quality control criteria for the PRACTICAL Consortium cohort were reported previously^26^.

### Selection of SNP-SNP interaction pairs

**Fig. 1:**
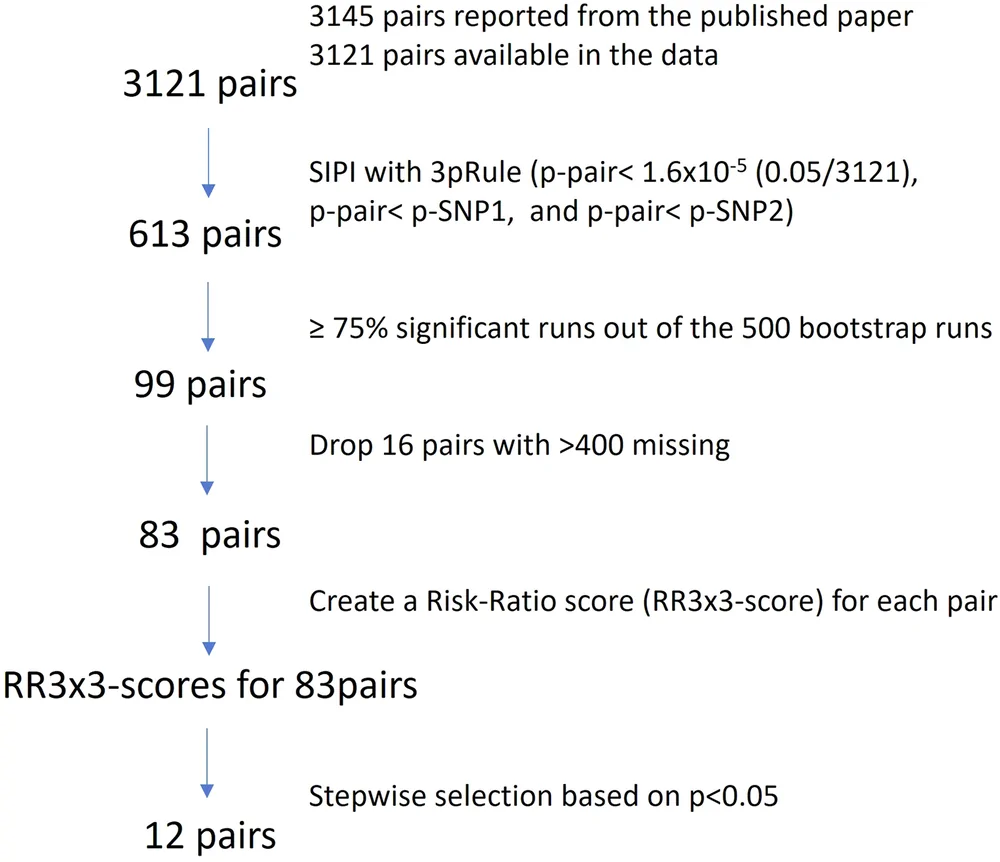
Process of selecting SNP-SNP interaction pairs based on the UK discovery dataset. SIPI SNP Interaction Pattern Identifier approach, 3pRule the 3 p-value rule, RR3x3 Risk Ratio score based on 3×3 genotype pair combinations; stepwise selection is based on logistic regression.

Our previous study identified 3145 SNP-SNP pairs significantly associated with PCa aggressiveness^8^. Among them, 3121 pairs were available in the PRACTICAL OncoArray SNP dataset (Fig. 1). The SNP Interaction Pattern Identifier (SIPI) approach was utilized to test SNP-SNP interaction pairs in this study^27^. SIPI is a machine learning method to test 45 interaction models for each SNP pair by considering three key factors: model structure, inheritance mode, and coding direction. In order to increase accuracy for detecting SNP-SNP interactions, the 2-stage 3pRule+bootstrap approach is suggested^28^. Thus, we applied the 2-stage 3pRule+bootstrap approach for selecting SNP-SNP interaction pairs in the UK set, the discovery set. In the 1^st^ stage, we applied the 3 p-value rule (3pRule), which is a modified rule to define significant SNP-SNP interaction pairs based on (1) p-pair of SNP1-SNP2 <p-pair-criterion (such as Bonferroni correction), (2) p-pair<p-main of SNP1, and (3) p-pair<p-main of SNP2. The p-pair is the p-value for an SNP-SNP interaction pair, and the p-main is the p-value of the 1st SNP or 2nd SNP in a pair. In this study, we applied the SIPI approach to get p-pairs. For testing SNP main effects, p-main values were obtained based on the minimum p-value of the 3 inheritance modes (dominant, recessive, and additive). In the 2nd stage, the bootstrap approach was applied to create datasets by resampling with replacement and with the same sample size as the original dataset. In the UK set, the p-pair-criterion was 1.6 × 10^−5^ ( = 0.05/3121) for the 3pRule set and was 1 × 10^−5^ for the 500 bootstrap runs. The “SIPI,” “eval3pRule”, and “boot3p_SIPI” R functions in the SIPI package were applied^27,29^.

### RR3x3 scoring system for SNP-SNP interaction pairs

For scoring SNP-SNP interaction pairs, we developed RR3x3 (Risk Ratio scoring based on 3×3 genotype pair combinations). This RR3x3 scoring especially benefits in maintaining valuable signals for high-risk genotype groups, usually with a small sample size. Each SNP has 3 potential genotypes (such as GG, GA, and AA). With a pair of SNPs, a 3-by-3 (3x3) table can be constructed with 9 genotype combinations (called pair-genotypes). While SIPI is powerful in detecting SNP-SNP interactions, the SIPI-interaction patterns simplify the 9-genotype combinations for a SNP pair into a few groups (such as 2 groups of high vs. low-risk). This feature hinders the SIPI-interaction pattern’s prediction accuracy. In order to preserve a complete risk profile of a SNP pair, we used the RR3x3 scoring approach instead. In this RR3x3 scoring method, a risk ratio of a genotype combination in a SNP pair (called pair-genotype, such as AA-GG and AG-GG) is calculated using an outcome percentage in a specific pair-genotype divided by the overall outcome percentage in the study. For this study, the outcome percentage is the percentage of PCa aggressiveness (aggr%), so the RR3x3 scores were calculated based on the aggr% for each pair-genotype divided by the overall aggr%. Pairs with a missing SNP in a pair were imputed based on the SNP main effect. For the pairs selected based on the 3pRule+ Bootstrap, the corresponding RR3x3 scores were calculated accordingly. Using the stepwise selection (*p* < 0.05) in the logistic model with the candidate pairs, the 12 pairs were selected. As shown in Eq. 1, PRS-12pair was calculated based on the sum of the RR3x3-scores of these selected 12 pairs.

1 $${{\rm{PRS}}}{\mbox{-}}12{{\rm{pair}}}= {{\rm{RR}}}3{{\rm{x}}}3{\mbox{-}}{{{\rm{score}}}}_{{{\rm{pair}}}1}+{{\rm{RR}}}3{{\rm{x}}}3 {\mbox{-}}{{{\rm{score}}}}_{{{\rm{pair}}}2}+\ldots +{{\rm{RR}}}3{{\rm{x}}}3{\mbox{-}}{{{\rm{score}}}}_{{{\rm{pair}}}12}$$

### Two PRSs with SNP main effects only

We are interested in comparing this PRS-12 pair with the 2 published PRSs, which only involved SNP main effects without considering SNP-SNP interactions. Two published PRSs with 166 SNPs^15^ and 290 SNPs^14^ were selected from the PGS catalog (PGS000742 and PGS003331)^30^. PGS000742 and PGS003331 were developed based on age at PCa diagnosis and age at aggressive PCa (Gleason score ≥7, PSA ≥ 10 ng/mL, T3-T4 stage, nodal metastases, or distant metastases), respectively, using time-to-event analyses with both controls and PCa cases. In these 2 published scores, 155 and 270 biallelic SNPs (PRS-m155 and PRS-m270) were tested in this study. The performance of the PRSs was evaluated based on the significance level of the selected PRS in logistic regression, adjusting for the first 7 principal components of population stratification as suggested by the PRACTICAL study^31^. For easy comparison, PRS-12pair, PRS-m155, and PRS-m270 were standardized. To leverage the benefits of the two PRSs, we created a PRS-KLK3int with 284 SNPs based on the weighted sum of PRS-12pair and PRS-m270. The weights were generated based on logistic regression.

In addition to testing numeric PRSs, we also tested categorical PRSs with 7 groups. For clinical usage, it is crucial to identify distinct high- and low-risk groups based on PRSs. Thus, we classified PCa patients into 7 groups based on PRS’s distribution in each study set. These 7 groups are the bottom 1%, bottom 1–10%, bottom 11–25%, middle 50%, top 11–25%, top 1–10%, and top 1%., which were defined based on the PRS’s percentiles of [0, 1), [1, 10), [10, 25), [25, 75), [75, 90), [90, 99), and [99, 100]. An open bracket “()” indicates the interval does not include the number itself, and a closed bracket “[]” includes the number itself. The significance was defined based on a *p*-value < 0.05. The Area Under the Receiver Operating Characteristic Curve (AUC) values for the models were calculated. Furthermore, we were interested in evaluating PRS performance by adding age at diagnosis.

## Results

### Detection of SNP-SNP interaction pairs for PRS-12pair

The procedure of selecting SNP pairs based on the UK set for PRS is listed in Fig. 1. Among the 3121 candidate SNP pairs, the 2-stage 3pRule+ bootstrap approach was applied to improve detection accuracy. By applying the 3pRule with p-pair<1.6 × 10^−5^ ( = 0.05/3121, Bonferroni correction) as the p-pair criterion, 613 pairs satisfied the 3pRule. In the 2nd stage, 99 pairs were satisfied with the 3pRule in ≥75% of 500 bootstrap datasets. These 99 pairs were grouped into the 6 clusters with 6 *KLK3* SNPs as a hub. The cluster is defined as several SNP pairs sharing a common or hub SNP. Among these 99 pairs, 50, 37, 15, 6, 3, and 2 pairs had rs17632542, rs2569735, rs1058205, rs174776, rs2292185, and rs266876 as the hub, respectively. Some pairs are involved with 2 *KLK3* SNPs. After excluding 16 pairs with >400 missing values, we generated the RR3X3-scores for the remaining 83 pairs for further analyses. After applying for stepwise selection with a *p*-value < 0.05 in the logistic model, 12 pairs were selected. Finally, PRS-12pair was calculated based on the sum of the RR3x3-scores of these 12 pairs. All 12 pairs were involved with *KLK3* SNPs and consist of only 15 SNPs. These 12 pairs and their gene information are listed in Supplementary Table 1.

### General results of 4 polygenic risk scores (PRSs) for PCa aggressiveness

The descriptive statistics of the 4 PRSs (PRS-12pair, PRS-m155, PRS-m270, PRS-KLK3int) are listed in Supplementary Table 2, and their distributions are shown in Supplementary Fig. 1. The distributions of the 2 PRS-main scores were close to a normal distribution. However, the distribution of PRS-12pair was skewed to the right (positively skewed), indicating that some men had a considerably high score of PCa aggressiveness. This is a desired feature for PRS to identify extremely high-risk groups. However, a small variation was observed for the low-value part of PRS-12pair; therefore, PRS-12pair may not be an ideal tool for distinguishing low-risk aggressive groups. These results inspired us to create a combined score, PRS-KLK3int, by integrating PRS-12pair and PRS-m270 to take advantage of both scores.

### Comparison of PRS-12pair with the 2 PRSs with SNP main effects only

For the 2 published PRSs, the associations between these 2 PRSs with SNP main effects only and PCa aggressiveness are listed in Supplementary Table 3. PRS-m155 and PRS-m270 can predict PCa aggressiveness for all 3 data sets (all p-values < 0.05). Specifically, we observed that PRS-m270 performed better than PRS-m155 for all 3 data sets, and these 2 PRSs performed better in the UK and EU sets than the UCA sets. Based on the adjusted results, the p-values for PRS-m155 were 7.5 × 10^–4^, 4.4 × 10^−4^, and 4.8 × 10^–8^ for the UK, UCA, and EU sets, respectively, while the p-values for PRS-m270 were 8.2 × 10^−7^, 3.3 × 10^–5^, and 9.4 × 10^–11^, respectively. In addition, the performance of PRS-12pair was better in terms of significance level than PRS-m155 and PRS-m270 in all 3 sets (Supplementary Table 3). The p-values for PRS-12pair were 5.4 × 10^–15^, 4.0 × 10^−8^, and 1.8 × 10^−13^ for the UK, UCA, and EU sets, respectively.

We tested whether PRS-12pair can further improve the performance of the PRS-m270. In the model with PRS-12pair and PRS-m270 (Supplementary Table 4), PRS-12pair performed better than PRS-m270 in all 3 sets. For the UK set, the p-values for PRS-m270 and PRS-12pair were 1.4 × 10^−4^ and 2.9 × 10^−12^. Similar conditions were observed in the UCA and EU sets. After adjusting PRS-m270 and the 7 principal components, PRS-12pair can still better predict PCa aggressiveness than PRS-m270 (*p* = 1.0 × 10^−10^ and 6.4 × 10^−11^ for the UCA and EU sets, respectively). Further, PRS-m270 was still significant (*p* = 1.9 × 10^−2^ and 9.6 × 10^−8^ for the UCA and EU sets, respectively).

### Performance evaluation of PRS-KLK3int (continuous score version)

The performance of PRS-KLK3int is shown in Supplementary Table 4. As expected, the PRS-KLK3int performed better than PRS-12pair and PRS-m270 alone for predicting PCa aggressiveness. The p-values for PRS-KLK3int were 3.5 × 10^−18^, 9 × 10^−14^, and 1.7 × 10^−20^ for the UK, UCA, and EU sets, respectively. As shown in Supplementary Table 5, the AUC values of PRS-KLK3int were 0.55, 0.58, and 0.59 after adjusting for the 7 principal components for the UK, UCA, and EU sets, respectively. Significant improvements were made by adding the 12 pairs into the PRS-m270 in all 3 sets. The *p*-values of the AUC comparison between PRS-KLK3int and PRS-m270 were 7.3 × 10^−5^, 0.004, and 0.051 for the UK, UCA, and EU sets, respectively.

### Performance evaluation of PRS-KLK3int (risk-group version)

**Fig. 2:**
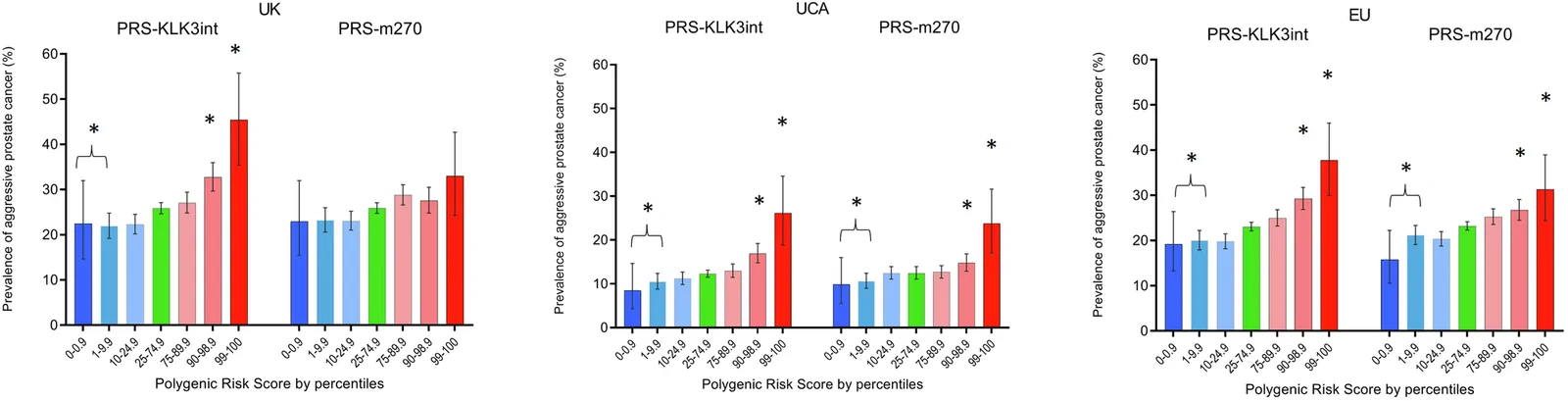
Prevalence of prostate cancer aggressiveness by 2 polygenic risk scores’ percentiles in the 3 study sets. UK: the United Kingdom set; UCA: the USA/Canada/Australia set; and EU: the set of other European countries. The stars (*) are for *p*-value < 0.05 compared with the middle 50% (25-74.9%) risk group based on the two-sided Wald chi-square tests in the logistic model adjusting for the 7 principal components of population stratification. The error bars represent 95% confidence intervals. The data can be found in Supplementary Table 6.

We further identified distinct high- and low-risk groups based on PRS-KLK3int, PRS-m270, and PRS-m155. For each study set, the PCa patients were classified into 7 groups based on PRS’s distribution. These 7 groups are bottom 1%, bottom 1–10%, bottom 11–25%, middle 50% (25–75%), top 11–25%, top 1–10%, and top 1%. PRS-KLK3int can distinguish high-risk PCa aggressiveness better than PRS-m270, and PRS-m155. The performance of risk classification of PCa aggressiveness for the UK, US, and EU sets in terms of PRS percentiles is listed in Fig. 2 and Supplementary Table 6.

In the UK set, PRS-KLK3int can significantly distinguish the top 1% and top 1–10% from the majority of the middle 50% group (Table 1 and Supplementary Tables 6 and 7). Based on PRS-KLK3int, the prevalence of PCa aggressiveness for the middle 50% group was 25.9%. PCa aggressiveness prevalence for the top 1% group (45.5%) was significantly higher than that in the middle 50% group (OR = 2.38, *p* = 2.2 × 10^−5^). This highlights that this PRS-KLk3int is able to categorize the top 1% of PCa patients at ~2-fold higher risk compared with the normal risk group. The top 1–10% group also had a significantly higher PCa aggressiveness prevalence than those in the middle 50% group (32.8% vs. 25.9%, OR = 1.4, *p* = 2.2 × 10^−5^). For detecting the low-risk groups, PRS-KLK3int did not perform well in distinguishing the extremely low score group (bottom 1%), but it can significantly distinguish the bottom 10% (OR = 0.81, *p* = 0.010). For the UK set, PRS-m155 and PRS-m270 cannot distinguish the top 1%, top 1–10%, and bottom 1% compared with the middle 50% group. However, PRS-m155 can distinguish the bottom 1–10% vs the middle 50% (OR = 0.81, *p* = 0.012), but PRS-m270 cannot.

**Table 1:** Comparisons of genetic-guided risk groups based on the polygenic risk scores (PRSs) for the 3 study sets

Study set ^a^	Top 1% vs. middle 50%		Top 1–10% vs. middle 50%		Bottom 1% vs. middle 50%		Bottom 1–10% vs. middle 50%		Bottom 0–10% vs. middle 50%	
	OR (95% CI)^b^	*p*-value	OR (95% CI)^b^	*p*-value	OR (95% CI)^b^	*p*-value	OR (95% CI)^b^	*p*-value	OR (95% CI)^b^	*p*-value
UK										
PRS-m155	1.38 (0.92, 2.08)	0.120	1.04 (0.89, 1.22)	0.595	1 (0.65, 1.54)	0.984	**0.81 (0.69, 0.95)**	**0.012**	**0.83 (0.71, 0.97)**	**0.017**
PRS-m270	1.40 (0.94, 2.10)	0.101	1.09 (0.94, 1.27)	0.260	0.85 (0.54, 1.34)	0.482	0.86 (0.73, 1.01)	0.070	0.86 (0.74, 1.00)	0.056
PRS-KLK3int	**2.38 (1.60, 3.56)**	**2.2x10**^**−5**^	**1.40 (1.20, 1.63)**	**2.2x10**^**−5**^	0.83 (0.52, 1.34)	0.448	**0.80 (0.68, 0.95)**	**0.012**	**0.81 (0.68, 0.95)**	**0.010**
UCA										
PRS-m155	**2.06 (1.39, 3.07)**	**0.0004**	1.12 (0.94, 1.33)	0.216	0.77 (0.44, 1.34)	0.352	0.89 (0.74, 1.07)	0.216	0.88 (0.73, 1.05)	0.151
PRS-m270	**2.17 (1.46, 3.22)**	**1.2x10**^**-4**^	**1.22 (1.03, 1.44)**	**0.025**	0.78 (0.45, 1.36)	0.382	0.83 (0.69, 1.01)	0.057	**0.83 (0.69, 0.99)**	**0.041**
PRS-KLK3int	**2.56 (1.71, 3.82)**	**4.3x10**^**−6**^	**1.45 (1.22, 1.72)**	**2.5×10**^**−5**^	0.65 (0.35, 1.21)	0.175	0.83 (0.68, 1.02)	0.073	**0.81 (0.67, 0.99)**	**0.036**
EU										
PRS-m155	1.4 (1, 1.96)	0.052	1.12 (0.99, 1.28)	0.078	**0.41 (0.25, 0.67)**	**0.0003**	0.89 (0.78, 1.02)	0.097	**0.84 (0.74, 0.96)**	**0.008**
PRS-m270	**1.53 (1.09, 2.14)**	**0.013**	**1.23 (1.09, 1.40)**	**0.001**	**0.61 (0.40, 0.93)**	**0.022**	0.87 (0.76, 1.00)	0.051	**0.85 (0.74, 0.96)**	**0.012**
PRS-KLK3int	**2.07 (1.48, 2.90)**	**2.1×10**^**−5**^	**1.38 (1.21, 1.57)**	**1.1×10**^**−6**^	0.78 (0.52, 1.17)	0.233	**0.82 (0.71, 0.95)**	**0.009**	**0.82 (0.71, 0.94)**	**0.005**

^a^ The United Kingdom (UK), USA/Canada/Australia (UCA), and other European countries (EU) sets.

^b^ Adjusted odds ratio (95% confidence interval) with a reference group using the middle 50% group. All models had a PRS and the 7 principal components of population stratification. Significant results with *p*-value < 0.05 based on the two-sided Wald chi-square tests in the logistic model were bold.

The performance of PRS-KLK3int in terms of PCa aggressiveness risk classification in the UCA and EU sets is similar to the UK set. For the UCA and EU sets, PRS-KLK3int can significantly distinguish the top 1% and top 1–10% from the majority of the middle 50% group. For PRS-KLK3int in the UCA set, PCa aggressiveness prevalence was 26.2%, 16.9%, and 12.3% for those in the top 1%, top 1–10% and middle 50% groups, respectively (Supplementary Table 7). PCa aggressiveness prevalence for the top 1% and top 1–10% groups was significantly higher than that in the middle 50% group (OR = 2.56, *p* = 4.3x10^-6^ for top 1%; OR = 1.45, *p* = 2.5x10^-5^ for top 1–10%). For PRS-KLK3int in the EU set, PCa aggressiveness prevalence was 37.8%, 29.3%, and 23.1% for those in the top 1%, top 1–10%, and middle 50% groups, respectively. PCa aggressiveness prevalence for the top 1% and top 1–10% groups was significantly higher than those in the middle 50% group (OR = 2.07, *p* = 2.1×10^-5^ for top 1%; OR = 1.38, *p* = 1.1x10^-6^ for top 1–10%). Similarly to the UK set, PRS-m270 and PRS-m155 performance was inferior to PRS-KLK3int in the UCA and EU sets. For the UCA set, PRS-m270 could distinguish the top 1% and top 1–10% versus the middle 50% for PCa aggressiveness (OR = 2.17, *p* = 1.2x10^-4^; OR = 1.22, *p* = 0.025, respectively). This similar trend of PRS-m270 can be observed in the EU set. PCa aggressiveness prevalence was significantly higher for the top 1% and top 1–10% compared with the middle 50% (OR = 1.53, *p* = 0.013; OR = 1.23, *p* = 0.001, respectively). For PRS-m155, PCa aggressiveness prevalence was significantly higher for the top 1% (OR = 2.06, *p* = 0.0004) in the UCA set and was significantly lower for the bottom 1% (OR = 0.41, *p* = 0.0003) in the EU set compared with the middle 50%. These results demonstrate that adding 12 pairs to PRS-m270 can significantly increase prediction accuracy for PCa aggressiveness, especially for the extremely high-risk group.

### PRS-KLK3int and age at PCa diagnosis for predicting PCa aggressiveness

We evaluated PRS-KLK3int performance by adding age at PCa diagnosis, a known risk factor of PCa aggressiveness, in models. As shown in Supplementary Table 8, age at diagnosis is positively associated with PCa aggressiveness for all 3 sets (UK: *p* = 5.0x10^–7^; UCA: *p* = 8.1x10^−46^; EU: *p* = 4.5x10^−116^). The age at PCa diagnosis was positively correlated with the risk of PCa aggressiveness. After adjusting age at diagnosis in the models, PRS-KLK3int remained highly significant (UK: *p* = 8.0x10^–17^; UCA: *p* = 2.7x10^–8^; EU: *p* = 5.8x10^–14^). Adding age at PCa diagnosis to the models increased the AUC values significantly for the UCA and EU sets but not the UK set (Supplementary Table 5). The AUC values were 0.56, 0.64, and 0.65 for the UK, UCA, and EU, respectively.

## Discussion

In this study, we developed PRS-KLK3int, could significantly predict PCa aggressiveness susceptibility more accurately than the other 3 PRSs alone (PRS-12pair, PRS-m270, and PRS-m155) when we evaluated the 3 large study sets (UK, UCA, and EU). To our knowledge, this is the first validated PRS involved SNP-SNP interactions for PCa aggressiveness. The major component of this score, PRS-12pair, contains 12 *KLK3* interaction pairs with only 15 SNPs. Notably, PRS-12pair outperformed the two other PRSs (PRS-m155 and PRS-m270) for predicting PCa aggressiveness. The PRS-KLK3int can improve distinguishing PCa patients with an extremely high risk (the top 1% and top 1–10%) and the bottom 10% compared with the middle-risk group of PCa aggressiveness. The p-values of PRS-KLK3int for comparing the top 1% with the middle-risk group are in a range of 4.3 × 10^−6^ to 2.2 × 10^−5^ for the 3 study sets. However, PRS-KLK3int did not perform well in differentiating the extremely low-risk group (bottom 1%), but can significantly predict the low-risk group in the bottom 10% based on its distribution. These findings demonstrated that PRS-KLK3int combines the advantages of both scores of PRS-12pair and PRS-m270, allowing it to differentiate between high- and low-risk groups of PCa aggressiveness.

The PRS-12 pair contains 15 SNPs in 13 genes, including *KLK3*. Among them, there are 3 *KLK3* SNPs (rs17632542, rs2569735, and rs174776). The genes linked to the 12 *KLK3* SNP pairs exhibit biological functions associated with PCa aggressiveness. *KLK3* is a gene that codes for PSA, which is a serine protease only secreted by the prostate gland^32^. In previous GWA studies, rs17632542 in *KLK3* was associated with various PCa phenotypes, including PSA level, PCa risk, aggressiveness, and Gleason score^21,33^. In addition, SNP-SNP interactions involving several *KLK3* SNPs (including rs17632542, rs2569735, and rs174776) can significantly predict PCa aggressiveness^8^. A meta-analysis study reported a significant association between rs174776 and PCa risk^34^. Among other genes in PRS-12pair, most genes are involved in PCa and other cancers, such as *SMAD3, CCND1, EPHB1, DEPTOR*, *JAZF1, PDCD6IP*, and *TPM1:LACTB*. *SMAD3* is overexpressed in advanced prostate tumors and regulates the expression of enzymes involved in the angiogenesis pathway^35,36^. Moreover, rs35874463 in *SMAD3* can predict height^37^, which is one of the known risk factors for PCa aggressiveness^38^. *CCND1 and KLK3* are also included in the 8-gene expression signature to predict the survival of PCa patients. This gene signature is based on circulating tumor cells, and its performance is better than most clinical variables^39^. *EPHB1* encodes a receptor tyrosine kinase involved in cell growth, differentiation, and apoptosis^40^, and its overexpression in prostate tumors suggests it may act as a potential oncogene^41^. In addition, expression of *DEPTOR* in prostate tumors was downregulated and correlated positively with PCa progression^42^. Low expression of *DEPTOR* promotes cell proliferation, survival, migration, and invasion, supporting its role as a potential tumor suppressor^43,44^. Regarding *JAZF1*, it is associated with PCa cell growth and response to polyamine-targeted therapies^45^. Moreover, the *JAZF1* rs849140 variant correlates with height^46^, a known risk factor for prostate cancer risk and aggressiveness. As for *PDCD6IP*, previous studies reported a positive correlation between the expression of *PDCD6IP* and Gleason Score^47^. *PDCD6IP* expression was significantly different in prostate malignant tissues when compared to indolent and normal prostate tissues^48^. rs2729786 is located in the intergenic region between *TPM1* and *LACTB*. *TPM1* was identified as one of 27 genes for a PCa progression gene signature^49^. This evidence highlights the potential biological role of genes linked to the 12 *KLK3* SNP pairs in PCa aggressiveness.

Although both PRS-m270 and PRS-m155 are significantly associated with PCa aggressiveness, it is important to discuss why these two PRSs showed a protective effect (OR < 1.0) on PCa aggressiveness. Several large-scale longitudinal studies have shown that men diagnosed with PCa at an older age have a higher risk of PCa-specific mortality, a terminal phenotype of PCa aggressiveness^16–18^. Our study results also indicated that age at PCa diagnosis is positively associated with PCa aggressiveness across all three sets. Consequently, a higher score of PRS-m155 and PRS-m270 suggests a higher chance of a younger age at PCa diagnosis, which may correspond to a lower risk of PCa aggressiveness.

We developed a promising PRS-KLK3int by integrating 12 *KLK3* SNP-SNP interaction pairs, which can be used in clinical settings as a valuable adjunct beyond existing tools primarily based on tumor gene expression (Decipher, Prolaris, and Oncotype-DX). This is because PRS-KLK3int can be used to classify high-risk PCa patients before biological onset, which will be beneficial for a large group of PCa patients under active surveillance. It has been shown that approximately 60% of low-risk PCa patients are under active surveillance^50^. Moreover, genetic scores can also play a crucial role in alleviating PCa patients’ anxiety stemming from the uncertainty of long-term PCa outcomes. Studies have shown that PCa patients’ anxiety and depression are common and are significantly associated with poor prognosis (such as PC progression and survival), even after treatments based on longitudinal studies^51,52^. The strength of this study lies in its rigorous study design and large discovery and validation datasets, which ensure the reliability and validity of the PRS findings. As shown in Fig. 1, the development of the PRS-12pair was through a solid process, including (1) the selection of candidate 3145 SNP pairs based on our previous solid study^8^, (2) application of SIPI for testing SNP-SNP interactions^27^, (3) the 3pRule+bootstrap SNP pair selection process to reduce false positivity and (4) the RR3x3 scoring system based on the risk of 9 genotype combinations of a SNP pair. The processes 2–4 were developed by our team.

While these study findings are promising, we are aware that there are some limitations. First, PRS-KLK3int, developed in this study to predict PCa aggressiveness, was derived from PCa patients with European ancestry. Thus, its applicability to other racial and ethnic groups remains uncertain and warrants further investigation. Notably, men with African ancestry are known to have a higher risk of incidence, aggressiveness, and poor outcomes than those with European ancestry^53,54^, underscoring the importance of validating and potentially adapting this genetic score in diverse populations. Second, the 12 SNP-SNP interaction pairs were selected based on SNP-SNP interactions using candidate genes in 4 major pathways^8^. A broader or genome-wide search level will be necessary to enhance the PRS of PCa aggressiveness. Third, while PRS-KLK3int shows promising results in terms of statistical significance, its AUC performance remains limited, indicating that it is not suitable for standalone use. However, it can be added to the existing tools to improve prediction accuracy. Finally, this study focused on genetic profiles without considering other factors, such as gene expression; therefore, its application in clinical settings needs further evaluation.

In conclusion, our findings highlight the PRS-KLK3int as a more effective tool for predicting PCa aggressiveness. This PRS supports risk stratification and personalized care to improve patient outcomes. In summary, PRS-KLK3 interaction effectively leverages interaction effects among biologically relevant SNPs to improve risk prediction for PCa aggressiveness. Its unique ability to predict PCa aggressiveness or progression before biological onset offers a valuable complement to existing tools in clinical settings. By refining genetic risk stratification, this model has the potential to support early decision-making in clinical practice. Future studies should explore different models of genome-wide interactions and extend validation to various populations to enhance applicability.

## Supplementary information

Supplemental Information

## Data Availability

The data used in this project are available from the Prostate Cancer Association Group to Investigate Cancer Associated Alterations in the Genome Consortium (PRACTICAL Consortium, https://practical.icr.ac.uk/), but restrictions apply to the availability of these data. Data access requests should be directed to practical@icr.ac.uk.

## Code availability

The SIPI function in the SIPI R package can be downloaded from the GitHub website^29^.
