# Supplementary material for: Polygenic risk score with KLK3 SNP-SNP interaction pairs for predicting prostate cancer aggressiveness
Source: Commun Med (Lond). 2026 May 28;6:451. doi: 10.1038/s43856-026-01645-z (PMC13493922; doi:10.1038/s43856-026-01645-z)
Supplement: Supplementary file 1 [file 43856_2026_1645_MOESM1_ESM.pdf]

Supplementary Table 1. List of 12 SNP-SNP interaction pairs (with 15 SNPs) and their gene information in the UK discovery set

SNP1	SNP2	Gene for SNP1	Gene for SNP2	SNP1 Maj/min allele (MAF)[a]	SNP2 Maj/min allele (MAF)[a]	SNP1 Chr: position[b]	SNP2 Chr: position[b]
rs2729786	rs17632542	*TPM1:LACTB*	*KLK3*	C/A (0.101)	A/G (0.055)	15: 63401605	19: 51361757
rs35874463	rs17632542	*SMAD3*	*KLK3*	A/G (0.059)	A/G (0.055)	15: 67457698	19: 51361757
rs527832	rs17632542	*WTAPP1*	*KLK3*	G/A (0.121)	A/G (0.055)	11: 102701884	19: 51361757
rs599190	rs17632542	*CCND1*	*KLK3*	G/A (0.195)	A/G (0.055)	11: 69429136	19: 51361757
rs6538566	rs17632542	*NDUFA12*	*KLK3*	C/A (0.212)	A/G (0.055)	12: 95296558	19: 51361757
rs6767950	rs17632542	*EPHB1*	*KLK3*	T/C (0.162)	A/G (0.055)	3: 134478108	19: 51361757
rs6874411	rs174776	*CDH12*	*KLK3*	A/C (0.437)	G/A (0.122)	5: 22661301	19: 51359852
rs7005380	rs2569735	*DEPTOR*	*KLK3*	G/A (0.358)	G/A (0.129)	8: 120953873	19: 51364269
rs7078775	rs17632542	*GPAM*	*KLK3*	A/C (0.105)	A/G (0.055)	10: 113912997	19: 51361757
rs7196117	rs17632542	*NUP93-DT*	*KLK3*	T/C (0.171)	A/G (0.055)	16: 56749994	19: 51361757
rs849140	rs17632542	*JAZF1*	*KLK3*	G/A (0.409)	A/G (0.055)	7: 28183702	19: 51361757
rs9838500	rs17632542	*PDCD6IP*	*KLK3*	A/G (0.163)	A/G (0.055)	3: 33928468	19: 51361757

[a] Major/minor allele; MAF: minor allele frequency

[b] Chr: chromosome; position is based on the Genome Reference Consortium Human Build 37 (GRCh37)

Supplementary Table 2. Descriptive statistics of the polygenic risk scores (PRS) with main effects only and their associations with prostate cancer aggressiveness by 3 study sets

**score**	**set**[a]	**n**	**mean± SD**[b]	**(min, max)**[b]		**Crude OR (95% CI)**[c]	**p-value**[c]		**adj. OR (95% CI)**[c,d]	**p-value**[c]
PRS-m155	UK	10836	0± 1	(-4.01, 3.76)		0.93(0.89-0.97)	$8.8x10^{-4}$		0.93 (0.89-0.97)	$7.5x10^{-4}$
	UCA	14348	0± 1	(-4.28, 4.87)		0.91(0.87-0.96)	$4.1x10^{-4}$		0.92 (0.87-0.96)	$4.4x10^{-4}$
	EU	16584	0± 1	(-3.63, 4.21)		0.91(0.88-0.94)	$2.8x10^{-7}$		0.90 (0.87-0.94)	$4.8x10^{-8}$
PRS-m270	UK	10836	0± 1	(-3.68, 3.84)		0.90 (0.86-0.94)	$9.4x10^{-7}$		0.90 (0.86-0.94)	$8.2x10^{-7}$
	UCA	14348	0± 1	(-3.84, 4.03)		0.90 (0.85-0.94)	$2.7x10^{-5}$		0.90 (0.85-0.94)	$3.3x10^{-5}$
	EU	16584	0± 1	(-3.59, 3.80)		0.89 (0.86-0.92)	$1.0x10^{-9}$		0.89 (0.86-0.92)	$9.4x10^{-11}$
PRS-12pair	UK	9811	0± 1	(-0.66, 11.25)		1.18 (1.13, 1.23)	$5.4x10^{-15}$		1.18 (1.13-1.23)	$5.4x10^{-15}$
	UCA	12957	0± 1	(-0.80, 7.67)		1.18 (1.13, 1.24)	$4.6x10^{-13}$		1.18 (1.13-1.24)	$4.0x10^{-8}$
	EU	15141	0± 1	(-0.68, 12.71)		1.15 (1.11, 1.19)	$5.4x10^{-15}$		1.15 (1.11-1.19)	$1.8x10^{-13}$
PRS-KLK3int	UK	9811	0± 0.19	(-0.39, 1.89)		2.73 (2.18, 3.43)	$3.9x10^{-18}$		2.74 (2.18, 3.44)	$3.5x10^{-18}$
	UCA	12957	0± 0.19	(-0.41, 1.29)		2.56 (2.00, 3.28)	$7.1x10^{-14}$		2.57 (2.00, 3.29)	$9.1x10^{-14}$
	EU	15141	0± 0.19	(-0.41, 2.03)		2.39 (1.99, 2.88)	$3.2x10^{-20}$		2.42 (2.01, 2.92)	$1.7x10^{-20}$

[a] The United Kingdom (UK), USA/Canada/Australia (UCA), and other European countries (EU) sets
[b] Standard deviation; min: minimum; max: maximum
[c] odds ratio and 95% confidence interval are per 1 standard deviation of the score. P-values were based on the two-sided Wald chi-square tests in the logistic models.
[d] adjusted for the first 7 principal components of population stratification

Supplementary Table 3. Performance of polygenic risk scores (PRS) for predicting prostate cancer aggressiveness by study sites

		UK[a] (discovery set)			UCA[a] (1st validation set)			EU[a] (2nd validation set)	
	n	OR (95% CI)[b]	p-value[b]	n	OR (95% CI)[b]	p-value[b]	n	OR (95% CI)[b]	p-value[b]
PRS-m155	10836	0.93 (0.89-0.97)	$7.5x10^{-4}$	14348	0.92 (0.87-0.96)	$4.4x10^{-4}$	16584	0.90 (0.87-0.94)	$4.8x10^{-8}$
PRS-m270	10836	0.90 (0.86-0.94)	$8.2x10^{-7}$	14348	0.90 (0.85-0.94)	$3.3x10^{-5}$	16584	0.89 (0.86-0.92)	$9.4x10^{-11}$
PRS-12pair	9811	1.18 (1.13-1.23)	$5.4x10^{-15}$	12957	1.18 (1.13-1.24)	$4.0x10^{-8}$	15141	1.15 (1.11-1.19)	$1.8x10^{-13}$

[a] The United Kingdom (UK), USA/Canada/Australia (UCA), and other European countries (EU) sets

[b] Adjusted odds ratio (95% confidence interval) per 1 standard deviation generated based on a model with a PRS and the 7 principal components of population stratification. P-values were based on the two-sided Wald chi-square tests in the logistic models.

Supplementary Table 4. Performance of combined PRS for predicting prostate cancer aggressiveness

			UK[a] (discovery set)			UCA[a] (1st validation set)			EU[a] (2nd validation set)	
Model		n	OR (95% CI)[b]	p-value[b]	n	OR (95% CI)[b]	p-value[b]	n	OR (95% CI)[b]	p-value[b]
Model-1	PRS-m270	9811	0.91 (0.87, 0.96)	$1.4x10^{-4}$	12957	0.94 (0.89, 0.99)	$1.9x 10^{-2}$	15141	0.9 (0.87, 0.94)	$9.6x 10^{-8}$
	PRS-12pair		1.16 (1.12, 1.21)	$2.9x10^{-12}$		1.17 (1.11, 1.22)	$1.0x10^{-10}$		1.12 (1.09, 1.17)	$6.4x10^{-11}$
Model-2[c]	PRS-KLK3int	9811	1.21 (1.16, 1.26)	$3.5x10^{-18}$	12957	1.19 (1.14, 1.25)	$9.1x10^{-14}$	15141	1.18 (1.14, 1.22)	$1.7x 10^{-20}$

[a] The United Kingdom (UK), USA/Canada/Australia (UCA), and other European countries (EU) sets

[b] Adjusted odds ratio (95% confidence interval) per 1 standard deviation. All models were adjusted for the 7 principal components of population stratification. P-values were based on the two-sided Wald chi-square tests in the logistic models.

[c] PRS-KLK3int is a score which combines PRS-12pair and PRS-m270

Supplementary Table 5. Area Under the Receiver Operating Characteristic Curve (AUC) values of the polygenic risk scores (PRSs)

Set [a]	Sample size	Model 1: PRS-m270	Model 2: PRS-KLK3int	Model 3: PRS-KLK3int + Age at Dx	Compare AUC for Models 2 vs. 1	Compare AUC for Models 3 vs. 2
		AUC	AUC	AUC	p-value[b]	p-value[b]
UK	9811	0.537	0.551	0.557	7.3×10^{-5}	0.267
UCA	12950	0.568	0.579	0.642	0.004	2.8×10^{-18}
EU	15140	0.582	0.586	0.647	0.051	1.1×10^{-30}

[a] The United Kingdom (UK), USA/Canada/Australia (UCA), and other European countries (EU) sets.
[b] P-values were based on the two-sided DeLong test.

Supplementary Table 6. Prostate cancer aggressiveness proportions by the percentiles of PRS-KLK3int and PRS-m270

Group name	Bottom 1%	Bottom 1-10%	Bottom 10-25%	Middle 50%	Top 10-25%	Top 1-10%	Top 1%
Group of PRS percentiles	0-0.9%	1-9.9%	10-24.9%	25-74.9%	75-89.9%	90-98.9%	99-100%
**PRS-KLK3int, UK**							
PCa aggr% (95% CI) [total N][b]	22.5 (14.6, 32) [98]	21.9 (19.2, 24.8) [885]	22.3 (20.2, 24.5) [1470]	25.9 (24.7, 27.1) [4904]	27.1 (24.8, 29.4) [1473]	32.8 (29.7, 36) [882]	45.5 (35.4, 55.8) [99]
OR (95% CI)[c]	0.83 (0.52, 1.34)	0.8 (0.68, 0.95)	0.82 (0.72, 0.94)	Reference	1.07 (0.93, 1.22)	1.40 (1.20, 1.63)	2.38 (1.60, 3.56)
p-value[c]	0.448	**0.012**	**0.006**		0.347	**2.2×10^{-5}**	**2.2×10^{-5}**
**PRS-KLK3int, UCA[a]**							
PCa aggr% (95% CI) [total N][b]	8.5 (4.3, 14.6) [130]	10.5 (8.8, 12.4) [1165]	11.2 (9.8, 12.7) [1945]	12.3 (11.5, 13.1) [6476]	13 (11.5, 14.5) [1946]	16.9 (14.8, 19.2) [1165]	26.2 (18.8, 34.6) [130]
OR (95% CI)[c]	0.65 (0.35, 1.21)	0.83 (0.68, 1.02)	0.9 (0.77, 1.06)	Reference	1.06 (0.91, 1.24)	1.45 (1.22, 1.72)	2.56 (1.71, 3.82)
p-value[c]	0.175	0.073	0.206		0.425	**2.5×10^{-5}**	**4.3×10^{-6}**
**PRS-KLK3int, EU[a]**							
PCa aggr% (95% CI) [total N][b]	19.2 (13.3, 26.4) [151]	20 (17.9, 22.2) [1364]	19.8 (18.2, 21.5) [2270]	23.1 (22.1, 24) [7571]	25 (23.2, 26.8) [2270]	29.3 (26.8, 31.7) [1364]	37.8 (30, 46) [151]
OR (95% CI)[c]	0.78 (0.52, 1.17)	0.82 (0.71, 0.95)	0.82 (0.73, 0.92)	Reference	1.12 (1.01, 1.25)	1.38 (1.21, 1.57)	2.07 (1.48, 2.90)
p-value[c]	0.233	**0.009**	**0.001**		**0.040**	**1.1×10^{-6}**	**2.1×10^{-5}**
**PRS-m270, UK[a]**							
PCa aggr% (95% CI) [total N][b]	22.9 (15.4, 32) [109]	23.2 (20.6, 26) [974]	23.1 (21, 25.2) [1626]	25.9 (24.8, 27.1) [5418]	28.8 (26.6, 31.1) [1625]	27.6 (24.8, 30.5) [975]	33 (24.3, 42.7) [109]
OR (95% CI)[c]	0.85 (0.54, 1.34)	0.86 (0.73, 1.01)	0.86 (0.75, 0.98)	Reference	1.16 (1.02, 1.31)	1.09 (0.94, 1.27)	1.4 (0.94, 2.1)
p-value[c]	0.482	0.070	**0.020**		**0.021**	0.260	0.101
**PRS-m270, UCA[a]**							
PCa aggr% (95% CI) [total N][b]	9.9 (5.5, 16) [142]	10.6 (9, 12.4) [1292]	12.4 (11.1, 13.9) [2154]	12.4 (11.1, 13.9) [7172]	12.7 (11.3, 14.2) [2153]	14.8 (12.9, 16.8) [1292]	23.8 (17.1, 31.6) [143]
OR (95% CI)[c]	0.78 (0.45, 1.36)	0.83 (0.69, 1.01)	1 (0.86, 1.16)	Reference	1.03 (0.89, 1.19)	1.22 (1.03, 1.44)	2.17 (1.46, 3.22)
p-value[c]	0.382	0.057	0.986		0.698	**0.025**	**1.2×10^{-4}**
**PRS-m270, EU[a]**							
PCa aggr% (95% CI) [total N][b]	15.8 (10.6, 22.2) [165]	21.2 (19.1, 23.3) [1493]	20.3 (18.8, 22) [2488]	23.2 (22.3, 24.1) [8293]	25.3 (23.6, 27) [2487]	26.7 (24.5, 29.1) [1492]	31.3 (24.4, 39) [166]
OR (95% CI)[c]	0.61 (0.4, 0.93)	0.87 (0.76, 1)	0.84 (0.75, 0.94)	Reference	1.12 (1.01, 1.24)	1.23 (1.09, 1.4)	1.53 (1.09, 2.14)
p-value[c]	**0.022**	0.051	**0.002**		**0.039**	**0.001**	**0.013**

[a] The United Kingdom (UK), USA/Canada/Australia (UCA), and other European countries (EU) sets
[b] N: sample size in the group, aggr%: percentage of prostate cancer aggressiveness
[c] Bold for p-value<0.05 were based on the two-sided Wald chi-square tests in the logistic models. Adjusted odds ratio (95% confidence interval) with a reference group using the middle 50% PRS. All models had a PRS and the 7 principal components of population stratification.

Supplementary Table 7. Prostate cancer aggressiveness proportions by the percentiles of PRS-m155

Group name	Bottom 1%	Bottom 1-10%	Bottom 10-25%	Middle 50%	Top 10-25%	Top 1-10%	Top 1%
Group of PRS percentiles	0-0.9%	1-9.9%	10-24.9%	25-74.9%	75-89.9%	90-98.9%	99-100%
**PRS-m155, UK**[a]							
PCa aggr% [total N][b]	25.7 [109]	22.1 [975]	25.5 [1621]	25.8 [5415]	27.8 [1642]	26.6 [966]	32.4 [108]
OR (95% CI)[c]	1 (0.65, 1.54)	0.81 (0.69, 0.95)	0.98 (0.86, 1.11)	Reference	1.11 (0.98, 1.25)	1.04 (0.89, 1.22)	1.38 (0.92, 2.08)
p-value[c]	0.984	**0.012**	0.762		0.112	0.595	0.120
**PRS-m155, UCA**[a]							
PCa aggr% [total N][b]	9.7 [144]	11.4 [1286]	11.7 [2171]	12.5 [7156]	13.4 [2145]	13.8 [1301]	22.8 [145]
OR (95% CI)[c]	0.77 (0.44, 1.34)	0.89 (0.74, 1.07)	0.93 (0.8, 1.08)	Reference	1.09 (0.94, 1.25)	1.12 (0.94, 1.33)	2.06 (1.39, 3.07)
p-value[c]	0.352	0.216	0.313		0.257	0.216	**0.0004**
**PRS-m155, EU**[a]							
PCa aggr% [total N][b]	11.5 [166]	21.6 [1486]	20.6 [2505]	23.6 [8281]	24.7 [2487]	25.4 [1493]	30.1 [166]
OR (95% CI)[c]	0.41 (0.25, 0.67)	0.89 (0.78, 1.02)	0.84 (0.76, 0.94)	Reference	1.08 (0.97, 1.2)	1.12 (0.99, 1.28)	1.4 (1, 1.96)
p-value[c]	**0.0003**	0.097	**0.003**		0.145	0.078	0.052

[a] The United Kingdom (UK), USA/Canada/Australia (UCA), and other European countries (EU) sets
[b] N: sample size in the group, aggr%: percentage of prostate cancer aggressiveness
[c] Bold for p-value<0.05 based on the two-sided Wald chi-square tests in the logistic models. Adjusted odds ratio (95% confidence interval) with a reference group using the middle 50% PRS. All models had a PRS and the 7 principal components of population stratification.

Supplementary Table 8. Model results of PRS-KLK3int by adjusting for age at prostate cancer diagnosis

	UK[a] (n=9811)		UCA[a] (n=12950)		EU[a] (n=15140)	
	**adj. OR (95% CI)**[b]	**p-value**[b]	**adj. OR (95% CI)**[b]	**p-value**[b]	**adj. OR (95% CI)**[b]	**p-value**[b]
Age at PCa diagnosis	1.02 (1.01, 1.02)	5.0×10^{-7}	1.05 (1.05, 1.06)	8.1×10^{-46}	1.06 (1.06, 1.07)	4.5×10^{-116}
PRS-KLK3int	2.63 (2.10, 3.31)	8.0×10^{-17}	2.06 (1.6, 2.66)	2.7×10^{-8}	2.09 (1.72, 2.53)	5.8×10^{-14}

[a]The United Kingdom (UK), USA/Canada/Australia (UCA), and other European countries (EU) sets
[b] Adjusted odds ratio (95% confidence interval) per 1 standard deviation. Model adjusted for the first 7 principal components of population stratification. P-values were based on the two-sided Wald chi-square tests in the logistic models.

Supplementary Figure 1. Distributions of four polygenic risk scores for predicting prostate cancer aggressiveness

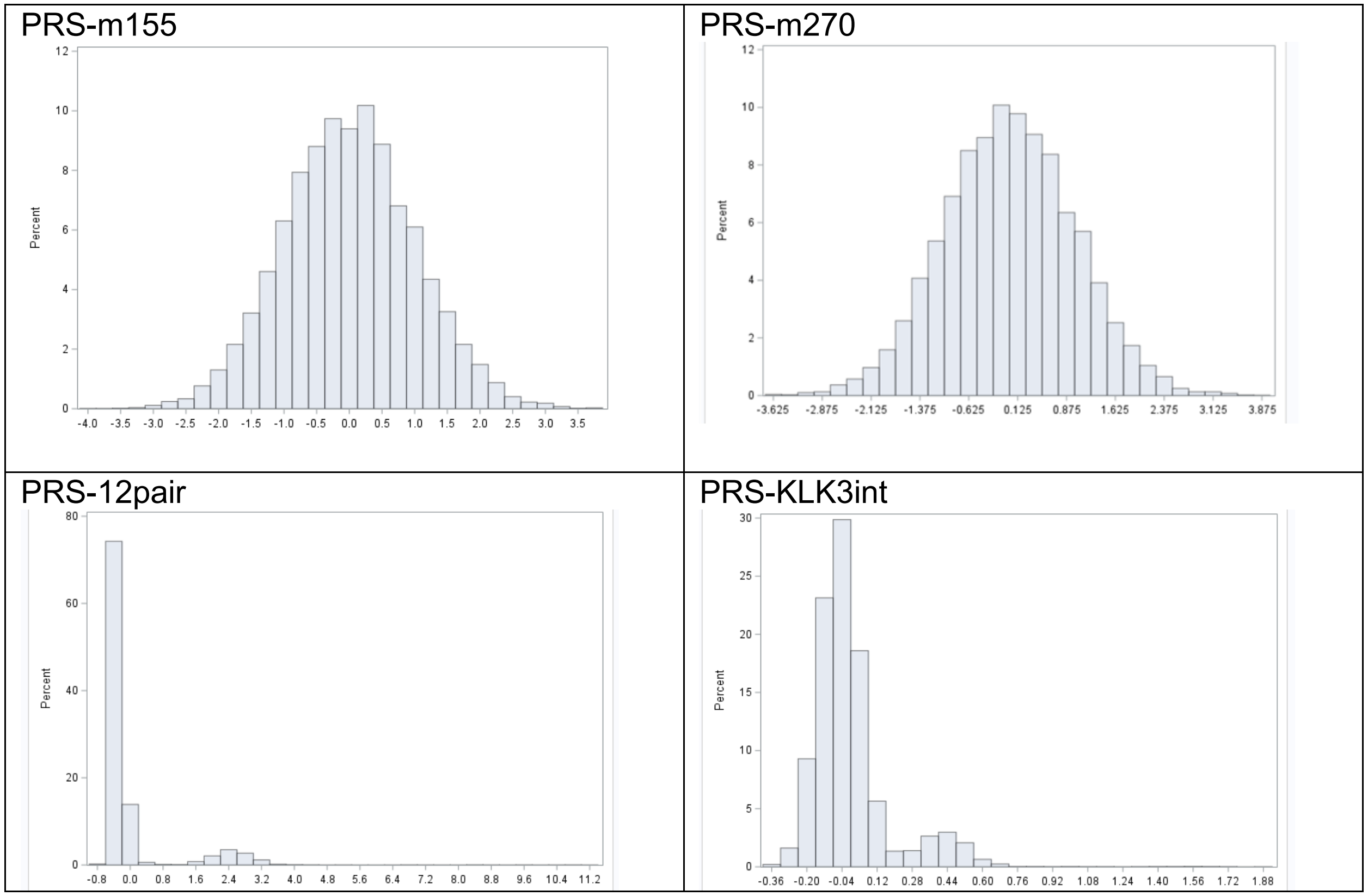

The Prostate Cancer Association Group to Investigate Cancer Associated Alterations in the Genome (PRACTICAL) CONSORTIUM (Supplementary authors in addition to those named in the main author list)

Information of the consortium can be found at http://practical.icr.ac.uk/

Additional members from the consortium are:

Artitaya Lophatananon,[1] Teuvo L. J. Tammela,[2,3] Anssi Auvinen,[4] Alison M. Dunning,[5] Maya Ghoussaini,[6] Suzanne Chambers,[7,8] Judith A. Clements,[9,10,11] Achala Shenali Fernando Vitharanage,[10,11] Lisa Horvath,[12,13] Gail P. Risbridger,[14,15] Wayne Tilley,[16] Markus Aly,[17,18,19] Tobias Nordström, [20,21] Jenny L. Donovan,[22] Freddie C. Hamdy,[23,24] Richard M. Martin,[22,25,26] Stig E. Bojesen,[27,28] Andreas Røder,[29] Hein V. Stroomberg,[29] Phyllis J. Goodman,[30] Ian M. Thompson Jr.,[31] Melissa C. Southey,[32,33,34] Graham G. Giles,[33,35,32] Roger L. Milne, [33,35,32] Niclas Håkansson,[36] Stephanie J. Weinstein,[37] Fredrick R. Schumacher,[38,39] Loic Le Marchand,[40] Xin Sheng,[41] Tim J. Key,[42] Edward Giovannucci,[43] Peter Kraft,[44] Bettina F. Drake,[45] Géraldine Cancel-Tassin,[46,47] Stephen Chanock,[37] Gerald L. Andriole,[48] Robert N. Hoover,[37] Mitchell J. Machiela,[37] Laura E. Beane Freeman,[37] Michael Borre,[49,50] Dominika Wokołorczyk,[51] Jan Lubinski,[51] Lovise Maehle,[52] Manuel Luedeke,[53] Thomas Schnoeller,[54] Neil E. Fleshner,[55] Antonio Finelli,[56] Sarah L. Kerns,[57] Harry Ostrer,[58] Antonio Gómez-Caamaño,[59] Laura Fachal,[60,61,62,63] Trinidad Dierssen-Sotos,[64,65] Gemma Castaño-Vinyals,[66,67,68,65] Antonio Alcaraz,[69] Robert Szulkin,[70,71] Martin Eklund,[72] Sara Lindström,[73] Meir Stampfer,[74] Paula Paulo,[75] Andreia Brandão,[75] Marta Cardoso,[75] Xin Gao,[76] Bernd Holleczek,[77] Ben Schöttker,[76] Chavdar Slavov,[78] Vanio Mitev,[79] Jeri Kim,[80] Yuan Chun Ding,[81] Linda Steele,[81] Davor Lessel,[82] Tomislav Kulis,[83] Matthew Parliament,[84,85] Aswin Abraham,[84] Steven Joniau,[86] Thomas Van den Broeck,[87,86] Manuela Gago-Dominguez,[88] Maria Elena Martinez,[89] David V. Conti,[41] Sara Benlloch,[90,91]

[1]Division of Population Health, Health Services Research and Primary Care, University of Manchester, Oxford Road, Manchester, M13 9PL, UK; [2]Department of Urology, Tampere University Hospital, FI-33521 Tampere, Finland; [3]Faculty of Medicine and Health Technology, Tampere University, FI-33100 Tampere, Finland; [4]Unit of Health Sciences, Faculty of Social Sciences, Tampere University, Tampere, Finland; [5]Centre for Cancer Genetic Epidemiology, Department of Oncology, University of Cambridge, Strangeways Laboratory, Worts Causeway, Cambridge, CB1 8RN, UK; [6]Open Targets, Wellcome Sanger Institute, Hinxton, Saffron Walden, CB10 1SA, UK; [7]Faculty of Health Sciences, Australian Catholic University, Australia; [8]Cancer Council Queensland, Fortitude Valley, QLD 4006, Australia; [9]School of Biomedical Sciences, Faculty of Health, Queensland University of Technology, Brisbane, Queensland, Australia; [10]Centre for Genomics and Personalised Health, Queensland University of Technology, Brisbane, Queensland, Australia; [11]Translational Research Institute, QUT, Woolloongabba, Brisbane, Queensland, Australia; [12]Chris O'Brien Lifehouse (COBLH), Camperdown, Sydney, NSW 2010, Australia; [13]Garvan Institute of Medical Research, Sydney NSW 2010, Australia; [14]Department of Anatomy and Developmental Biology, Biomedicine Discovery Institute, Monash University, Melbourne, Victoria 3800, Australia; [15]Prostate Cancer Translational Research Program, Cancer Research Division, Peter MacCallum Cancer Centre, Melbourne, VIC 3000, Australia; [16]Dame Roma Mitchell Cancer Research Laboratories, University of Adelaide, Adelaide, South Australia, Australia;

[17]Department of Medical Epidemiology and Biostatistics, Karolinska Institutet, SE-171 77 Stockholm, Sweden; [18]Department of Molecular Medicine and Surgery, Karolinska Institutet, Karolinska University Hospital, Solna, 171 76 Stockholm, Sweden; [19]Department of Urology, Karolinska University Hospital, Solna, 171 76 Stockholm, Sweden; [20]Department of Medical Epidemiology and Biostatistics, Karolinska Institutet, Stockholm, Sweden; [21]Department of Clinical Sciences at Danderyds Hospital, Karolinska Institutet, Stockholm, Sweden; [22]Population Health Sciences, Bristol Medical School, University of Bristol, BS8 2PS, UK; [23]Nuffield Department of Surgical Sciences, University of Oxford, Oxford, OX1 2JD, UK; [24]Faculty of Medical Science, University of Oxford, John Radcliffe Hospital, Oxford, UK; [25]National Institute for Health Research (NIHR) Biomedical Research Centre, University of Bristol, Bristol, BS8 1TH, UK; [26]Medical Research Council (MRC) Integrative Epidemiology Unit, University of Bristol, Bristol, BS8 2BN, UK; [27]Faculty of Health and Medical Sciences, University of Copenhagen, 2200 Copenhagen, Denmark; [28]Department of Clinical Biochemistry, Herlev and Gentofte Hospital, Copenhagen University Hospital, Herlev, 2200 Copenhagen, Denmark; [29]Copenhagen Prostate Cancer Center, Dept. of Urology, Copenhagen University Hospital - Rigshospitalet, Copenhagen, Denmark; [30]SWOG Statistical Center, Fred Hutchinson Cancer Research Center, Seattle, WA 98109, USA; [31]CHRISTUS Santa Rosa Hospital-Medical Center, San Antonio, Texas 78229, USA; [32]Precision Medicine, School of Clinical Sciences at Monash Health, Monash University, Clayton, Victoria 3168, Australia; [33]Cancer Epidemiology Division, Cancer Council Victoria, 200 Victoria Parade, East Melbourne, VIC, 3002, Australia; [34]Department of Clinical Pathology, The Melbourne Medical School, The University of Melbourne, Melbourne, VIC 3010, Australia; [35]Centre for Epidemiology and Biostatistics, Melbourne School of Population and Global Health, The University of Melbourne, Grattan Street, Parkville, VIC 3010, Australia; [36]Unit of Cardiovascular and Nutritional Epidemiology, Institute of Environmental Medicine, Karolinska Institutet, SE-171 77 Stockholm, Sweden; [37]Division of Cancer Epidemiology and Genetics, National Cancer Institute, NIH, Bethesda, Maryland, 20892, USA; [38]Department of Population and Quantitative Health Sciences, Case Western Reserve University, Cleveland, OH 44106-7219, USA; [39]Seidman Cancer Center, University Hospitals, Cleveland, OH 44106, USA; [40]Epidemiology Program, University of Hawaii Cancer Center, Honolulu, HI 96813, USA; [41]Center for Genetic Epidemiology, Department of Preventive Medicine, Keck School of Medicine, University of Southern California/Norris Comprehensive Cancer Center, Los Angeles, CA 90015, USA; [42]Cancer Epidemiology Unit, Nuffield Department of Population Health, University of Oxford, Oxford, OX3 7LF, UK; [43]Department of Epidemiology, Harvard School of Public Health, Boston, MA 02115, USA; [44]Program in Genetic Epidemiology and Statistical Genetics, Department of Epidemiology, Harvard School of Public Health, Boston, MA, USA; [45]Washington University School of Medicine, 660 S. Euclid Avenue, Campus Box 8242, St. Louis, MO 63110 , USA; [46]CeRePP, Tenon Hospital, F-75020 Paris, France; [47]Sorbonne Universite, GRC n°5 , AP-HP, Tenon Hospital, 4 rue de la Chine, F-75020 Paris, France; [48]The Washington University School of Medicine, 660 S. Euclid Avenue, Campus Box 8242, St. Louis, MO 63110 , USA; [49]Department of Urology, Aarhus University Hospital, Palle Juul-Jensen Boulevard 99, 8200 Aarhus N, Denmark; [50]Department of Clinical Medicine, Aarhus University, DK-8200 Aarhus N; [51]International Hereditary Cancer Center, Department of Genetics and Pathology, Pomeranian Medical University, 70-115 Szczecin, Poland; [52]Department of Medical Genetics, Oslo University Hospital, 0424 Oslo, Norway; [53]Genetikum, Wegenerstr. 15, D-89231 Neu-Ulm, Germany; [54]Department of Urology, University Hospital Ulm, Germany; [55]Dept. of Surgical Oncology, UHN Princess Margaret Cancer Centre, Toronto ON M5G 2M9,

Canada; [56]Division of Urology, Princess Margaret Cancer Centre, Toronto ON M5G 2M9, Canada; [57]The Medical College of Wisconsin, 8701 Watertown Plank Rd., Milwaukee, WI 53226, USA; [58]Department of Pathology, Albert Einstein College of Medicine, 1300 Morris Park Avenue, Ullman 817, Bronx, NY 10461, USA; [59]Department of Radiation Oncology, Complexo Hospitalario Universitario de Santiago, SERGAS, Santiago de Compostela 15706, Spain;[60]Centre for Cancer Genetic Epidemiology, Department of Public Health and Primary Care, University of Cambridge, Strangeways Research Laboratory, Cambridge, CB2 0SR, UK; [61]Fundación Pública Galega Medicina Xenómica, Santiago De Compostela, 15706, Spain; [62]Instituto de Investigación Sanitaria de Santiago de Compostela, Santiago de Compostela, 15706, Spain; [63]Centro de Investigación en Red de Enfermedades Raras (CIBERER), Spain; [64]University of Cantabria-IDIVAL, Santander, Spain; [65]CIBER Epidemiolog¡a y Salud Pública (CIBERESP), 28029 Madrid, Spain; [66]ISGlobal, Barcelona, Spain; [67]IMIM (Hospital del Mar Medical Research Institute), Barcelona, Spain; [68]Universitat Pompeu Fabra (UPF), Barcelona, Spain; [69]Department and Laboratory of Urology. Hospital Clínic. Institut d'Investigacions Biomèdiques August Pi i Sunyer (IDIBAPS), Universitat de Barcelona. Spain. C/Villarroel 170; 08036 Barcelona, Spain; [70]Department of Medical Epidemiology and Biostatistics, Karolinska Institutet, Stockholm; [71]SDS Life Science, Danderyd, Sweden; [72]Department of Medical Epidemiology and Biostatistics, Karolinska Institutet, 171 77 Stockholm, Sweden; [73]Department of Epidemiology, Health Sciences Building, University of Washington, Seattle, WA 98195, USA; [74]Channing Division of Network Medicine, Department of Medicine, Brigham and Women's Hospital/Harvard Medical School, Boston, MA 02184, USA; [75]Cancer Genetics Group, IPO Porto Research Center (CI-IPOP) / RISE@CI-IPOP (Health Research Network), Portuguese Oncology Institute of Porto (IPO Porto) / Porto Comprehensive Cancer Center, Porto, Portugal; [76]Division of Clinical Epidemiology and Aging Research, German Cancer Research Center (DKFZ), D-69120, Heidelberg, Germany; [77]Saarland Cancer Registry, 66119 Saarbrücken, Germany; [78]Department of Urology and Alexandrovska University Hospital, Medical University of Sofia, 1431 Sofia, Bulgaria; [79]Molecular Medicine Center, Department of Medical Chemistry and Biochemistry, Medical University of Sofia, Sofia, 2 Zdrave Str., 1431 Sofia, Bulgaria; [80]The University of Texas M. D. Anderson Cancer Center, Department of Genitourinary Medical Oncology, 1515 Holcombe Blvd., Houston, TX 77030, USA; [81]Department of Population Sciences, Beckman Research Institute of the City of Hope, 1500 East Duarte Road, Duarte, CA 91010, USA; [82]Institute of Human Genetics, University Medical Center Hamburg-Eppendorf, D-20246 Hamburg, Germany; [83]Department of Urology, University Hospital Center Zagreb, University of Zagreb School of Medicine , Zagreb, Croatia; [84]Department of Oncology, Cross Cancer Institute, University of Alberta, 11560 University Avenue, Edmonton, Alberta, Canada T6G 1Z2; [85]Division of Radiation Oncology, Cross Cancer Institute, 11560 University Avenue, Edmonton, Alberta, Canada T6G 1Z2; [86]Department of Urology, University Hospitals Leuven, Herestraat 49, Box 7003 41, BE-3000 Leuven, Belgium; [87]Molecular Endocrinology Laboratory, Department of Cellular and Molecular Medicine, KU Leuven, BE-3000, Belgium; [88]Health Research Institute of Santiago de Compostela Foundation (FIDIS), Cancer Genetics and Epidemiology Group, Santiago de Compostela, Spain; [89] University of California San Diego, Moores Cancer Center, Department of Family Medicine and Public Health, University of California San Diego, La Jolla, CA 92093-0012, USA; [90] Centre for Cancer Genetic Epidemiology, Department of Public Health and Primary Care, University of Cambridge, Strangeways Research Laboratory, Cambridge CB1 8RN, UK; [91]The Institute of Cancer Research, London, SM2 5NG, UK

Funding for the PRACTICAL consortium

http://practical.icr.ac.uk/

This work was supported by the Canadian Institutes of Health Research, European Commission's Seventh Framework Programme grant agreement n° 223175 (HEALTH-F2-2009-223175), Cancer Research UK Grants C5047/A7357, C1287/A10118, C1287/A16563, C5047/A3354, C5047/A10692, C16913/A6135, and The National Institute of Health (NIH) Cancer Post-Cancer GWAS initiative grant: No. 1 U19 CA 148537-01 (the GAME-ON initiative).

We would also like to thank the following for funding support: The Institute of Cancer Research and The Everyman Campaign, The Prostate Cancer Research Foundation, Prostate Research Campaign UK (now PCUK), The Orchid Cancer Appeal, Rosetrees Trust, The National Cancer Research Network UK, The National Cancer Research Institute (NCRI) UK. We are grateful for support of NIHR funding to the NIHR Biomedical Research Centre at The Institute of Cancer Research and The Royal Marsden NHS Foundation Trust.
The Prostate Cancer Program of Cancer Council Victoria also acknowledge grant support from The National Health and Medical Research Council, Australia (126402, 209057, 251533, , 396414, 450104, 504700, 504702, 504715, 623204, 940394, 614296,), VicHealth, Cancer Council Victoria, The Prostate Cancer Foundation of Australia, The Whitten Foundation, PricewaterhouseCoopers, and Tattersall's. EAO, DMK, and EMK acknowledge the Intramural Program of the National Human Genome Research Institute for their support.

Genotyping of the OncoArray was funded by the US National Institutes of Health (NIH) [U19 CA 148537 for ELucidating Loci Involved in Prostate cancer SuscEptibility (ELLIPSE) project and X01HG007492 to the Center for Inherited Disease Research (CIDR) under contract number HHSN268201200008I]. Additional analytic support was provided by NIH NCI U01 CA188392 (PI: Schumacher).

This study would not have been possible without the contributions of the following: Per Hall (COGS); Douglas F. Easton, Paul Pharoah, Kyriaki Michailidou, Manjeet K. Bolla, Qin Wang (BCAC), Andrew Berchuck (OCAC), Rosalind A. Eeles, Douglas F. Easton, Ali Amin Al Olama, Zsofia Kote-Jarai, Sara Benlloch (PRACTICAL), Georgia Chenevix-Trench, Antonis Antoniou, Lesley McGuffog, Fergus Couch and Ken Offit (CIMBA), Joe Dennis, Alison M. Dunning, Andrew Lee, and Ed Dicks, Craig Luccarini and the staff of the Centre for Genetic Epidemiology Laboratory, Javier Benitez, Anna Gonzalez-Neira and the staff of the CNIO genotyping unit, Jacques Simard and Daniel C. Tessier, Francois Bacot, Daniel Vincent, Sylvie LaBoissière and Frederic Robidoux and the staff of the McGill University and Génome Québec Innovation Centre, Stig E. Bojesen, Sune F. Nielsen, Borge G. Nordestgaard, and the staff of the Copenhagen DNA laboratory, and Julie M. Cunningham, Sharon A. Windebank, Christopher A. Hilker, Jeffrey Meyer and the staff of Mayo Clinic Genotyping Core Facility

Additional funding and acknowledgments from studies in PRACTICAL:

Aarhus
This study was supported by Innovation Fund Denmark, the Danish Cancer Society and The Velux Foundation (Veluxfonden). The Danish Cancer Biobank (DCB) is acknowledged for biological material.

AHS
This work was supported by the Intramural Research Program of the NIH, National Cancer Institute, Division of Cancer Epidemiology and Genetics (Z01CP010119).

ATBC
The ATBC Study is supported by the Intramural Research Program of the U.S. National Cancer Institute, National Institutes of Health, Department of Health and Human Services.

CCI
This work was awarded by Prostate Cancer Canada and is proudly funded by the Movember Foundation - Grant # D2013-36. The CCI group would like to thank David Murray, Razmik Mirzayans, and April Scott for their contribution to this work.

COH
SLN is partially supported by the Morris and Horowitz Families Endowed Professorship

COSM
COSM is part of the Swedish Infrastructure for Medical Population-based Life-course and Environmental Research (SIMPLER), which receives funding from the Swedish Research Council (grants VR 2017-00644, VR 2021-00160.This work was supported by the Swedish Cancer Foundation (grant CF 20 0864).

CPCS1/CPCS2
Department of Clinical Biochemistry, Herlev and Gentofte Hospital, Copenhagen University Hospital, Herlev Ringvej 75, DK-2730 Herlev, Denmark. We thank participants and staff of the Copenhagen General Population Study for their important contributions.

EPIC
The coordination of EPIC was financially supported by the European Commission (DG-SANCO) and the International Agency for Research on Cancer. The national cohorts (that recruited male participants) are supported by Danish Cancer Society (Denmark); German Cancer Aid, German Cancer Research Center (DKFZ), Federal Ministry of Education and Research (BMBF), Deutsche Krebshilfe, Deutsches Krebsforschungszentrum and Federal Ministry of Education and Research (Germany); the Hellenic Health Foundation (Greece); Associazione Italiana per la Ricerca sul Cancro-AIRC-Italy and National Research Council (Italy); Dutch Ministry of Public Health, Welfare and Sports (VWS), Netherlands Cancer Registry (NKR), LK Research Funds, Dutch Prevention Funds, Dutch ZON (Zorg Onderzoek Nederland), World Cancer Research Fund (WCRF), Statistics Netherlands (The Netherlands); Health Research Fund (FIS), PI13/00061 to Granada; , PI13/01162 to EPIC-Murcia), Regional Governments of Andalucía, Asturias, Basque Country, Murcia and Navarra, ISCIII RETIC (RD06/0020) (Spain); Swedish Cancer Society, Swedish Research Council and County Councils of Skåne and Västerbotten (Sweden); Cancer Research UK (14136 to EPIC-Norfolk; C8221/A29017 to EPIC-Oxford), Medical Research Council (1000143 to EPIC-Norfolk, MR/M012190/1 to EPIC-Oxford) (United Kingdom). For information on how to submit an application for gaining access to EPIC data and/or biospecimens, please follow the instructions at http://epic.iarc.fr/access/index.php"

ESTHER
The ESTHER study was supported by a grant from the Baden Württemberg Ministry of Science, Research and Arts. The ESTHER group would like to thank Hartwig Ziegler, Sonja Wolf, Volker Hermann, Heiko Müller, Karina Dieffenbach, Katja Butterbach for valuable contributions to the study.

FHCRC

The FHCRC studies were supported by grants R01-CA080122, R01-CA056678, R01-CA082664, and R01-CA092579, and K05-CA175147 from the US National Cancer Institute, National Institutes of Health, with additional support from the Fred Hutchinson Cancer Research Center (P30-CA015704). We thank all the individuals who participated in these studies.

Gene-PARE
The Gene-PARE study was supported by grants 1R01CA134444 from the U.S. National Institutes of Health , PC074201 and W81XWH-15-1-0680 from the Prostate Cancer Research Program of the Department of Defense and RSGT-05-200-01-CCE from the American Cancer Society. S.L.K. is supported by 1K07CA187546 from the U.S. National Cancer Institute.

HPFS
The Health Professionals Follow-up Study was supported by grants UM1CA167552, CA133891, CA141298, and P01CA055075. We are grateful to the participants and staff of the Physicians' Health Study and Health Professionals Follow-Up Study for their valuable contributions, as well as the following state cancer registries for their help: AL, AZ, AR, CA, CO, CT, DE, FL, GA, ID, IL, IN, IA, KY, LA, ME, MD, MA, MI, NE, NH, NJ, NY, NC, ND, OH, OK, OR, PA, RI, SC, TN, TX, VA, WA, WY.

IMPACT
The IMPACT study was funded by The Ronald and Rita McAulay Foundation, CR-UK Project grant (C5047/A1232), Cancer Australia, AICR Netherlands A10-0227, Cancer Australia and Cancer Council Tasmania, NIHR, EU Framework 6, Cancer Councils of Victorial and South Australia, Philanthropic donation to Northshore University Health System. We acknowledge support from the National Institute for Health Research (NIHR) to the Biomedical Research Centre at The Institute of Cancer Research and Royal Marsden Foundation NHS Trust. We acknowledge the IMPACT study steering committee, collaborating centres and participants.

IPO-Porto
The IPO-Porto study was funded by Fundação para a Ciência e a Tecnologia (FCT; UIDP/0076/2020, CEECINST/00091/2018, and 2021.03835.CEECIND) and by IPO-Porto Research Center (CI-IPOP-24-2015). We would like to express our gratitude to all patients and families who have participated in this study.

KULEUVEN
F.C. and S.J. are holders of grants from FWO Vlaanderen (G.0684.12N and G.0830.13N), the Belgian federal government (National Cancer Plan KPC_29_023), and a Concerted Research Action of the KU Leuven (GOA/15/017). TVDB is holder of a doctoral fellowship of the FWO.

MCC-Spain
The study was partially funded by the ""Accion Transversal del Cancer"", approved on the Spanish Ministry Council on the 11th October 2007, by the Instituto de Salud Carlos III-FEDER (PI08/1770, PI09/00773-Cantabria, PI11/01889-FEDER, PI12/00265, PI12/01270, PI12/00715, PI15/00069), by the Fundación Marqués de Valdecilla (API 10/09), by the Spanish Association Against Cancer (AECC) Scientific Foundation and by the Catalan Government DURSI grant 2009SGR1489. Samples: Biological samples were stored at the Parc de Salut MAR Biobank (MARBiobanc; Barcelona) which is supported by Instituto de Salud Carlos III FEDER (RD09/0076/00036). Also sample collection was supported by the Xarxa de Bancs de Tumors de Catalunya sponsored by Pla Director d'Oncologia de Catalunya (XBTC). ISGlobal acknowledges support from the Spanish Ministry of Science

and Innovation through the “Centro de Excelencia Severo Ochoa 2019-2023” Program (CEX2018-000806-S), and support from the Generalitat de Catalunya through the CERCA Program. We acknowledge the contribution from Esther Gracia-Lavedan in preparing the data. We thank all the subjects who participated in the study and all MCC-Spain collaborators.

MCCS
The Melbourne Collaborative Cohort Study (MCCS) cohort recruitment was funded by VicHealth and Cancer Council Victoria. The MCCS was further supported by Australian National Health and Medical Research Council grants 209057 and 396414 and by infrastructure provided by Cancer Council Victoria. Cases and their vital status were ascertained through the Victorian Cancer Registry. The Prostate Cancer Program of Cancer Council Victoria also acknowledge grant support from The National Health and Medical Research Council, Australia (126402, 209057, 251533, 396414, 450104, 504700, 504702, 504715, 623204, 940394, 614296,), VicHealth, Cancer Council Victoria, The Prostate Cancer Foundation of Australia, The Whitten Foundation, PricewaterhouseCoopers, and Tattersall’s.

MEC
The MEC was supported by NIH grants CA063464, CA054281, CA098758, and CA164973.

MOFFITT
The Moffitt group was supported by the US National Cancer Institute (R01CA128813, PI: J.Y. Park).

Oslo
CONOR was supported by grants from the Nordic Cancer Union, the Swedish Cancer Society (2012/823) and the Swedish Research Council (2014/2269). The authors wish to acknowledge the services of CONOR, the contributing research centres delivering data to CONOR, and all the study participants.

PCMUS
The PCMUS study was supported by the Bulgarian National Science Fund, Ministry of Education and Science (contract DOO-119/2009; DUNK01/2-2009; DFNI-B01/28/2012) with additional support from the Science Fund of Medical University - Sofia (contract 51/2009; 8I/2009; 28/2010;).

PHS
The Physicians’ Health Study was supported by grants CA34944, CA40360, CA097193, HL26490 and HL34595. We are grateful to the participants and staff of the Physicians’ Health Study and Health Professionals Follow-Up Study for their valuable contributions, as well as the following state cancer registries for their help: AL, AZ, AR, CA, CO, CT, DE, FL, GA, ID, IL, IN, IA, KY, LA, ME, MD, MA, MI, NE, NH, NJ, NY, NC, ND, OH, OK, OR, PA, RI, SC, TN, TX, VA, WA, WY.

PLCO
This PLCO study was supported by the Intramural Research Program of the Division of Cancer Epidemiology and Genetics, National Cancer Institute, NIH. The authors thank Drs. Christine Berg and Philip Prorok, Division of Cancer Prevention at the National Cancer Institute, the screening center investigators and staff of the PLCO Cancer Screening Trial for their contributions to the PLCO Cancer Screening Trial. We thank Mr. Thomas Riley, Mr. Craig Williams, Mr. Matthew Moore, and Ms. Shannon Merkle at Information Management Services, Inc., for their management of the data and Ms. Barbara O’Brien and staff at Westat, Inc. for their contributions to the PLCO Cancer Screening Trial. We also thank the PLCO study participants for their contributions to making this study possible.

PRAGGA
PRAGGA was supported by Programa Grupos Emergentes, Cancer Genetics Unit, CHUVI Vigo Hospital, Instituto de Salud Carlos III, Spain.
PRAGGA wishes to thank Victor Muñoz Garzón, Manuel Enguix Castelo, Sara Miranda Ponte, Carmen M Redondo, Manuel Calaza, Francisco Gude Sampedro, Joaquín González-Carreró and the staff of the Department of Pathology and Biobank of University Hospital Complex of Vigo, Instituto de Investigación Sanitaria Galicia Sur (IISGS), SERGAS, Vigo, Spain; Máximo Fraga, José Antúnez and the Biobank of University Hospital Complex of Santiago, Santiago de Compostela, Spain; and Maria Torres, Angel Carracedo and the Galician Foundation of Genomic Medicine.

PROCAP
PROCAP was supported by the Swedish Cancer Foundation (08-708, 09-0677).
We thank and acknowledge all of the participants in the PROCAP study. We thank Carin Cavalli-Björkman and Ami Rönnberg Karlsson for their dedicated work in the collection of data. Michael Broms is acknowledged for his skilful work with the databases. KI Biobank is acknowledged for handling the samples and for DNA extraction. We acknowledge The NPCR steering group: Pär Stattin (chair), Anders Widmark, Stefan Karlsson, Magnus Törnblom, Jan Adolfsson, Anna Bill-Axelson, Ove Andrén, David Robinson, Bill Pettersson, Jonas Hugosson, Jan-Erik Damber, Ola Bratt, Göran Ahlgren, Lars Egevad, and Roy Ehrnström.

PROFILE
We would like to acknowledge the support of the Ronald and Rita McAulay Foundation and Cancer Research UK. We also acknowledge support from the National Institute for Health Research (NIHR) to the Biomedical Research Centre at The Institute of Cancer Research and Royal Marsden Foundation NHS Trust. We acknowledge the Profile study steering committee and participants.

PROGReSS
This research was supported by Spanish Instituto de Salud Carlos III (ISCIII) funding, an initiative of the Spanish Ministry of Economy and Innovation partially supported by European Regional Development FEDER Funds (INT15/00070, INT16/00154, INT17/00133, INT20/00071, INT24/00023; DTS24/00083; PI22/00589, PI19/01424, PI16/00046; PI13/02030; PI10/00164), and through the Autonomous Government of Galicia (Consolidation and structuring program: IN607B) given to A.Vega. We would like to thank the patients for their contribution to the study.

ProMPT / ProtecT
ProtecT would like to acknowledge the support of The University of Cambridge, Cancer Research UK. Cancer Research UK grants [C8197/A10123] and [C8197/A10865] supported the genotyping team. We would also like to acknowledge the support of the National Institute for Health Research which funds the Cambridge Bio-medical Research Centre, Cambridge, UK. We would also like to acknowledge the support of the National Cancer Research Prostate Cancer: Mechanisms of Progression and Treatment (PROMPT) collaborative (grant code G0500966/75466) which has funded tissue and urine collections in Cambridge. We are grateful to staff at the Welcome Trust Clinical Research Facility, Addenbrooke's Clinical Research Centre, Cambridge, UK for their help in conducting the ProtecT study. We also acknowledge the support of the NIHR Cambridge Biomedical Research Centre, the NIHR HTA (ProtecT grant) and the NCRI / MRC (ProMPT grant) for help with the bio-repository. The UK Department of Health funded the ProtecT study through the NIHR Health Technology Assessment Programme (projects 96/20/06, 96/20/99). The ProtecT trial and its linked ProMPT and CAP (The Cluster Randomized Trial of PSA Testing

for Prostate Cancer) studies are supported by Department of Health, England; Cancer Research UK grant number C522/A8649, C11043/A4286, C18281/A8145, C18281/A11326, and C18281/A15064, Medical Research Council of England grant number G0500966, ID 75466 and The NCRI, UK. The epidemiological data for ProtecT were generated though funding from the Southwest National Health Service Research and Development. DNA extraction in ProtecT was supported by USA Dept of Defense award W81XWH-04-1-0280, Yorkshire Cancer Research and Cancer Research UK. The authors would like to acknowledge the contribution of all members of the ProtecT study research group. The views and opinions expressed therein are those of the authors and do not necessarily reflect those of the Department of Health of England. The bio-repository from ProtecT is supported by the NCRI (ProMPT) Prostate Cancer Collaborative and the Cambridge BMRC grant from NIHR. We acknowledge support from the National Cancer Research Institute (National Institute of Health Research (NIHR) Collaborative Study: “Prostate Cancer: Mechanisms of Progression and Treatment (PROMPT)” (grant G0500966/75466). We thank the National Institute for Health Research, Hutchison Whampoa Limited, the Human Research Tissue Bank (Addenbrooke’s Hospital), and Cancer Research UK. The authors would like to thank those men with prostate cancer and the subjects who have donated their time and their samples to the Cambridge Biorepository, which were used in this research. We also would like to acknowledge to support of the research staff in S4 who so carefully curated the samples and the follow-up data (Jo Burge, Marie Corcoran, Anne George, and Sara Stearn)." Richard Martin was supported by a Cancer Research UK Programme Grant (C18281/A19169) and the National Institute for Health Research Bristol Nutrition Biomedical Research Centre based at University Hospitals Bristol NHS Foundation Trust and the University of Bristol.

QLD
The QLD research is supported by The National Health and Medical Research Council (NHMRC) Australia Project Grants [390130, 1009458] and NHMRC Career Development Fellowship, Cancer Australia PdCCRS and Cancer Council Queensland funding to J Batra. The QLD team would like to acknowledge and sincerely thank the urologists, pathologists, data managers and patient participants who have generously and altruistically supported the QLD cohort.

SEARCH
SEARCH is funded by a programme grant from Cancer Research UK [C490/A10124] and supported by the UK National Institute for Health Research Biomedical Research Centre at the University of Cambridge. The University of Cambridge has received salary support in respect of PP from the NHS in the East of England through the Clinical Academic Reserve.

SFPCS
SFPCS was funded by California Cancer Research Fund grant 99-00527V-10182

SNP Prostate Ghent
The study was supported by the National Cancer Plan, financed by the Federal Office of Health and Social Affairs, Belgium.

SPAG
Manchester Cancer Research Centre and MRC – Medical Research Council for PhD studentship work, MUG - Male Uprising in Guernsey, Hope for Guernsey and Wessex Medical Research for research support

STHM2
STHM2 was supported by grants from The Strategic Research Programme on Cancer (StratCan), Karolinska Institutet; the Linné Centre for Breast and Prostate Cancer (CRISP, number 70867901), Karolinska Institutet; The Swedish Research Council (number K2010-

70X-20430-04-3) and The Swedish Cancer Society (numbers 11-0287 and 11-0624); Stiftelsen Johanna Hagstrand och Sigfrid Linnérs minne; Swedish Council for Working Life and Social Research (FAS), number 2012-0073. The authors acknowledge the Karolinska University Laboratory, Aleris Medilab, Unilabs and the Regional Prostate Cancer Registry for performing analyses and help to retrieve data. Carin Cavalli–Björkman and Britt-Marie Hune for their enthusiastic work as research nurses. Astrid Björklund for skilful data management. We wish to thank the BBMRI.se biobank facility at Karolinska Institutet for biobank services.

SWOG-PCPT / SWOG-SELECT
PCPT/SELECT is funded by Public Health Service grants U10CA37429 and 5UM1CA182883 from the National Cancer Institute. The authors thank the site investigators and staff and, most importantly, the participants from PCPT who donated their time to this trial.

TAMPERE
The Tampere (Finland) study was supported by the Academy of Finland (251074), The Finnish Cancer Organisations, Sigrid Juselius Foundation, and the Competitive Research Funding of the Tampere University Hospital (X51003). The PSA screening samples were collected by the Finnish part of ERSPC (European Study of Screening for Prostate Cancer). TAMPERE would like to thank Riina Liikanen, Liisa Maeaettaenen and Kirsi Talala for their work on samples and databases.

Toronto
Prostate Cancer Canada Movember Discovery Grant (D2013-17) to RJH; Canadian Cancer Society Research Institute Career Development Award in Cancer Prevention (2013-702108) to RJH

UKGPCS
UKGPCS would also like to thank the following for funding support: The Institute of Cancer Research and The Everyman Campaign, The Prostate Cancer Research Foundation, Prostate Research Campaign UK (now Prostate Cancer UK), The Orchid Cancer Appeal, The National Cancer Research Network UK, The National Cancer Research Institute (NCRI) UK. We are grateful for the support of NIHR funding to the NIHR Biomedical Research Centre at The Institute of Cancer Research and The Royal Marsden NHS Foundation Trust. UKGPCS should also like to acknowledge the NCRN nurses, data managers and Consultants for their work in the UKGPCS. UKGPCS would like to thank all urologists and other persons involved in the planning, coordination, and data collection of the study. KM and AL were in part supported from the NIHR Manchester Biomedical Research Centre

ULM
The Ulm group received funds from the German Cancer Aid (Deutsche Krebshilfe).

WUGS
WUGS would like to thank the following for funding support: The Anthony DeNovi Fund, the Donald C. McGraw Foundation, and the St. Louis Men's Group Against Cancer.
